# Supplementary material for: Systematic Qualitative and Quantitative Analyses of Wenxin Granule via Ultra-High Performance Liquid Chromatography Coupled with Ion Mobility Quadrupole Time-of-Flight Mass Spectrometry and Triple Quadrupole–Linear Ion Trap Mass Spectrometry
Source: Molecules. 2022 Jun 6;27(11):3647. doi: 10.3390/molecules27113647 (PMC9181919; doi:10.3390/molecules27113647)
Supplement: Supplementary file 1 [file molecules-27-03647-s001.zip › molecules-1729015-supplementary.pdf]

<*Molecules*>

Supplementary Materials

Systematic Qualitative and Quantitative Analyses of Wenxin Granule by Ultra-High Performance Liquid Chromatography Coupled with Ion Mobility Quadrupole Time-of-Flight Mass Spectrometry and Triple Quadrupole/Linear Ion-Trap Mass Spectrometry

Yueguang Mi [1,†], Wandi Hu [1,†], Weiwei Li [1,†], Shiyu Wan [2], Xiaoyan Xu [1], Meiyu Liu [1], Hongda Wang [1], Quanxi Mei [2], Qinhua Chen [2], Yang Yang [2], Boxue Chen [1], Meiting Jiang [1], Xue Li [1], Wenzhi Yang [1,*] and Dean Guo [1,3]

[1] State Key Laboratory of Component-Based Chinese Medicine, Tianjin Key Laboratory of TCM Chemistry and Analysis, Tianjin University of Traditional Chinese Medicine, 10 Poyanghu Road, Jinghai, Tianjin 301617, China; miyueguang@163.com (Y.M.); hwdcrown@163.com (W.H.); lww11413@163.com (W.L.); xxy_0421@163.com (X.X.); lmydz1999@163.com (M.L.); 17862987156@163.com (H.W.); cbx1026tju@163.com (B.C.); jiangmeiting21@163.com (M.J.); tjdxsyx@163.com (X.L.)

[2] Shenzhen Baoan Authentic TCM Therapy Hospital, Shenzhen 518101, China; wanshiyuaaa@163.com (S.W.); meiquanxi@163.com (Q.M.); cqh77@163.com (Q.C.); yangyanghb@outlook.com (Y.Y.)

[3] Shanghai Research Center for Modernization of Traditional Chinese Medicine, National Engineering Laboratory for TCM Standardization Technology, Shanghai Institute of Materia Medica, Chinese Academy of Sciences, 501 Haike Road, Shanghai 201203, China; gda5958@163.com (D.G.)

[*] Correspondence: wzyang0504@tjutcm.edu.cn, Tel.: +86-022-5979-1833

[†] These authors contributed equally to this work.

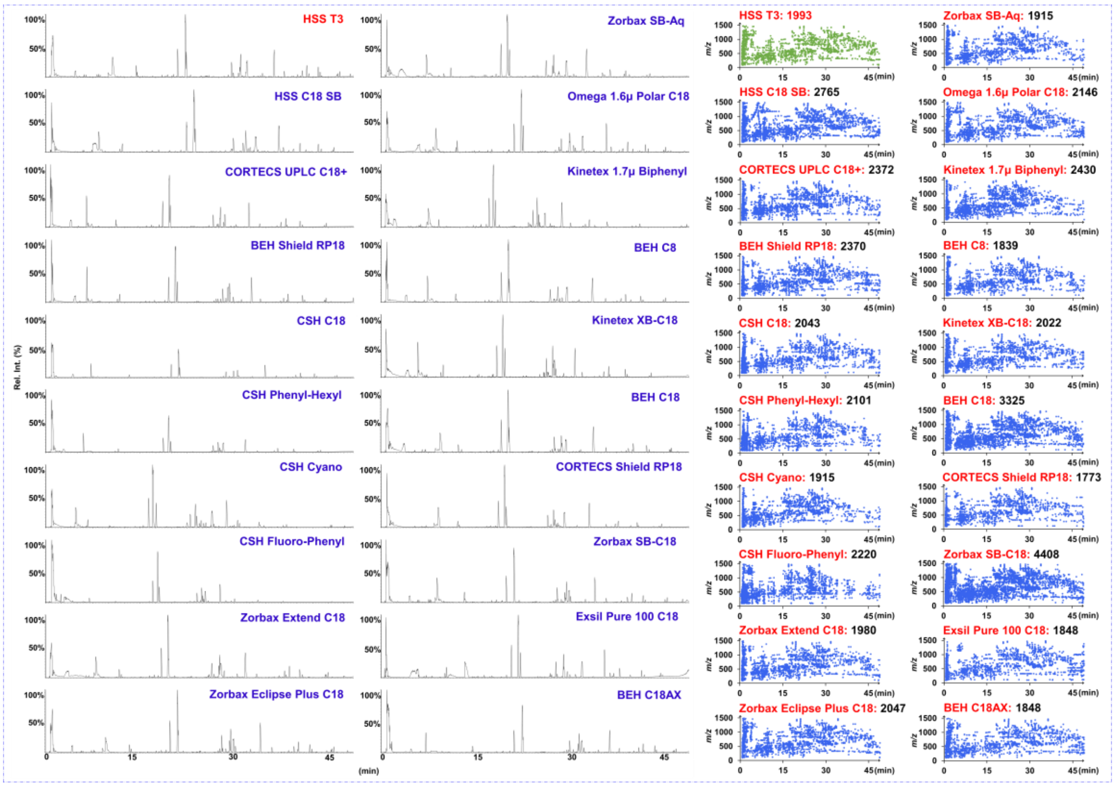

Figure S1. Selection of the stationary phase for the ultra-high performance liquid chromatography (UHPLC) separation of the multicomponents from WXG. The left shows the base peak intensity (BPI) chromatograms obtained on 20 candidate columns; the right is the scatter plot of the components resolved by both MS and chromatographic separation on each column.

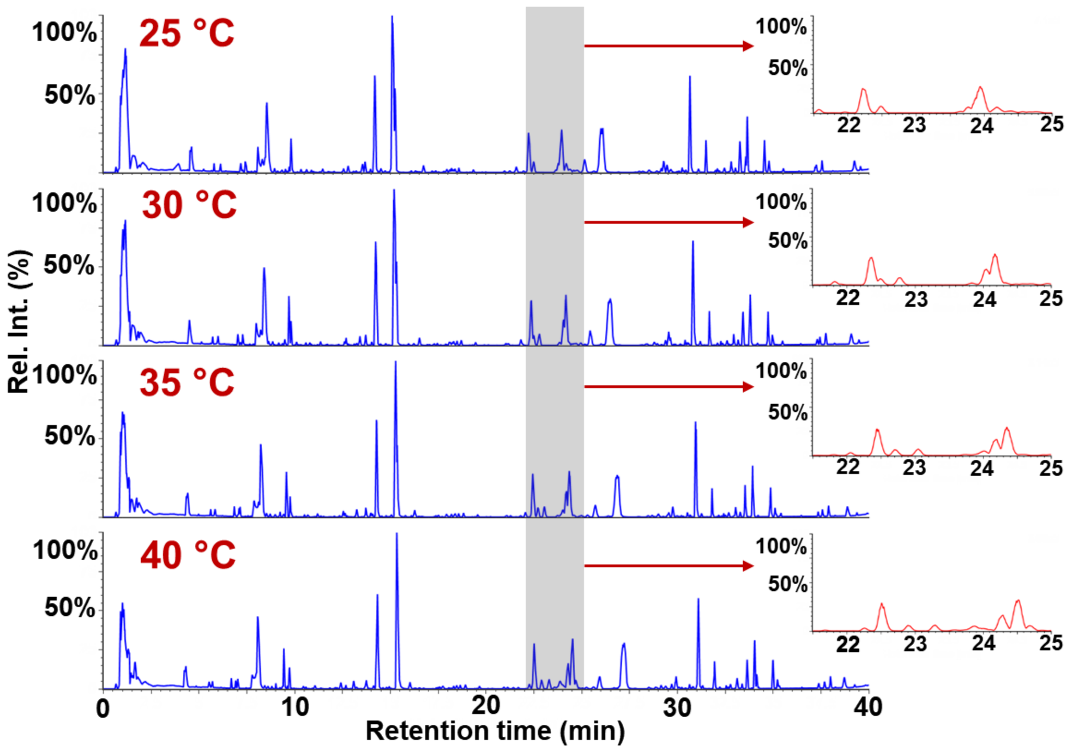

Figure S2. Optimization of the column temperature on the selected HSS T3 column for the separation of the multicomponents from WXG.

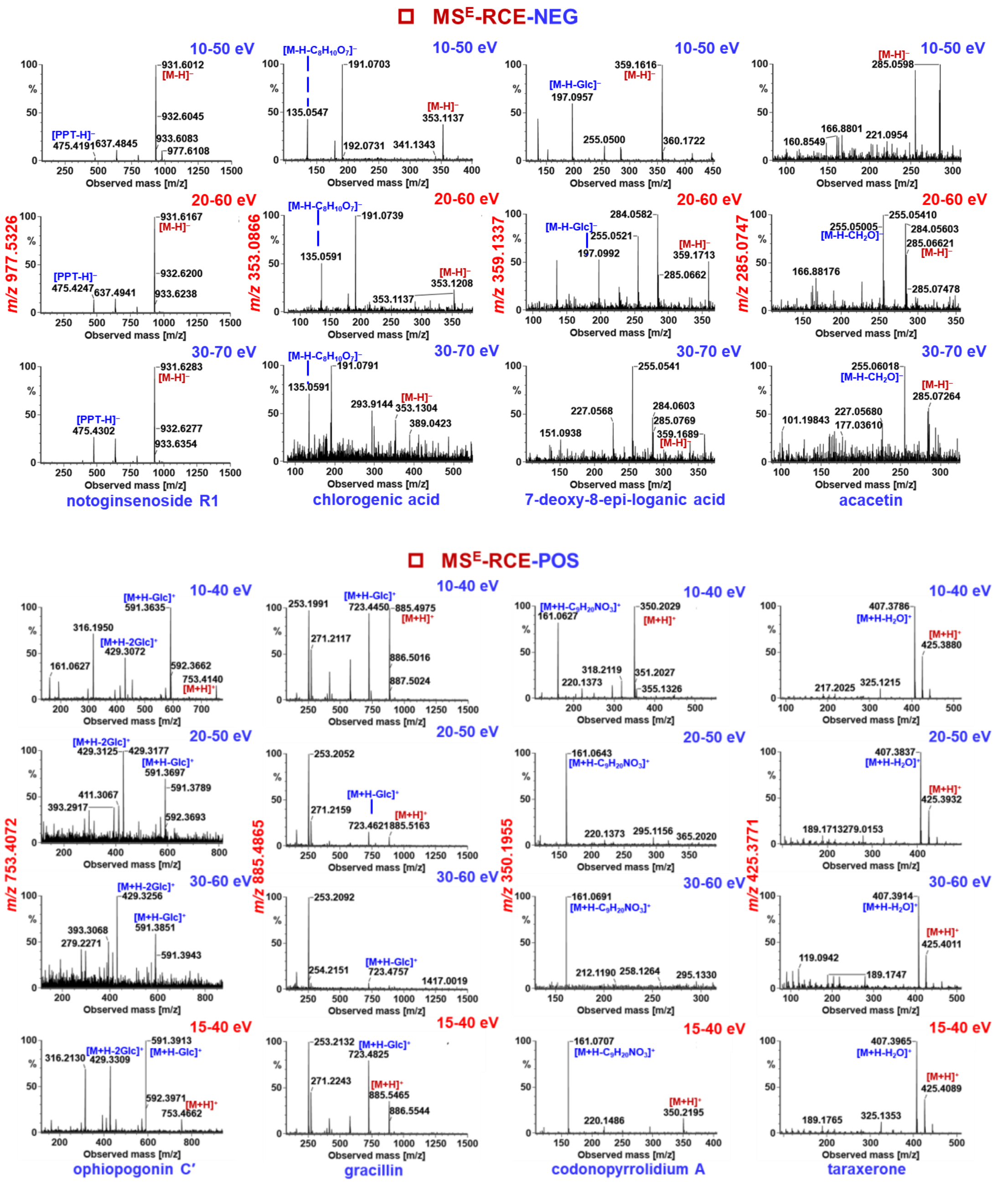

Figure S3. Optimization of ramp collision energy (RCE) for the HDMS^E approach in both the negative (NEG) and positive (POS) modes using the representative compounds from WXG.

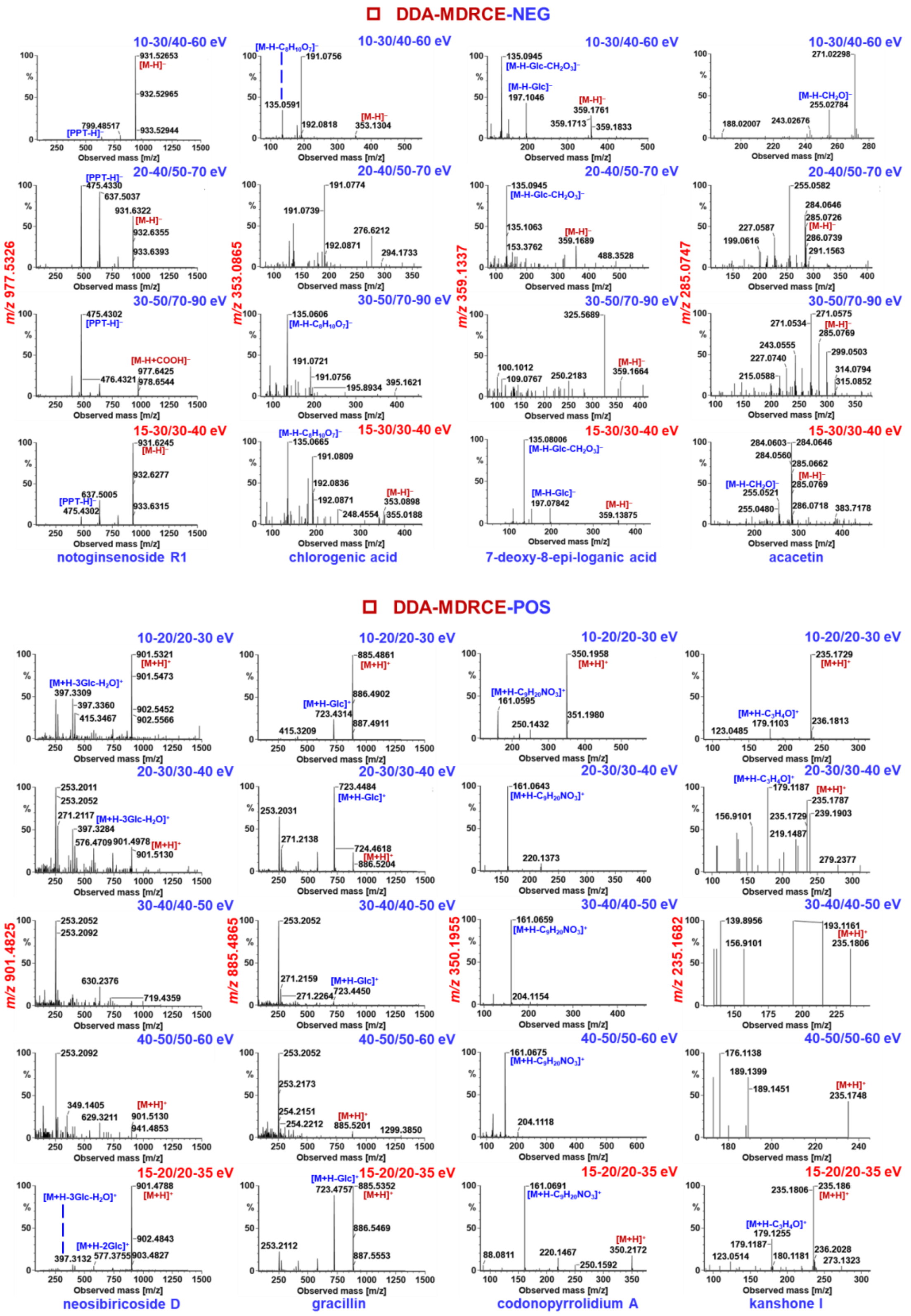

Figure S4. Optimization of mass-dependent ramp collision energy (MDRCE) for the HDDDA approach in both the negative (NEG) and positive (POS) modes using the representative compounds from WXG.

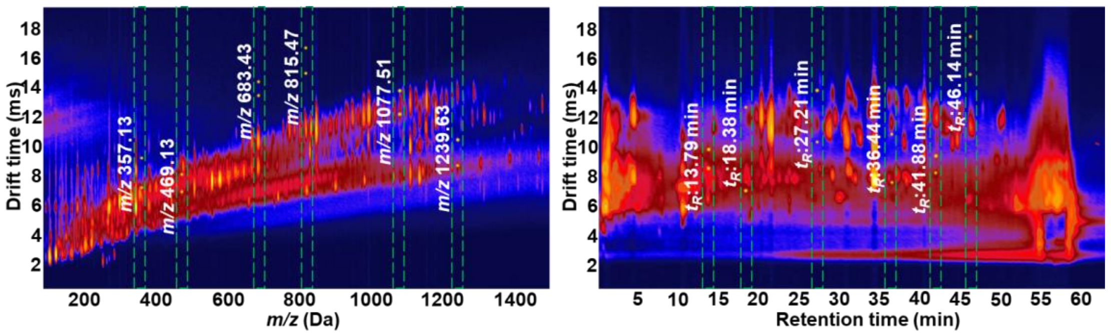

Figure S5. Drift time VS *m/z* showing the separation of isomers, and drift time VS t_R showing the separation of the co-eluting components from WXG.

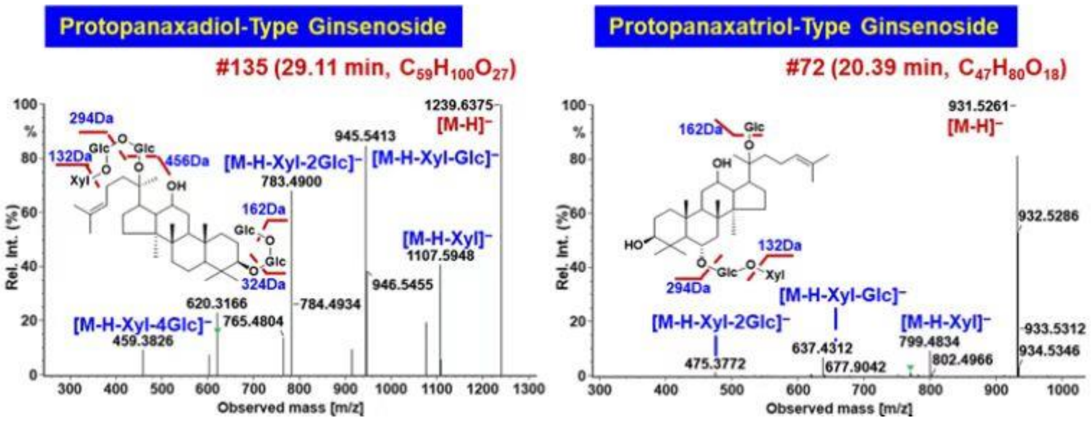

Figure S6. Annotation of the CID-MS^2 spectra of the representative protopanaxadiol (PPD)-, and protopanaxatriol (PPT)-ginsenosides from WXG by UNIFI^{TM}, identified by comparison with the reference compounds.

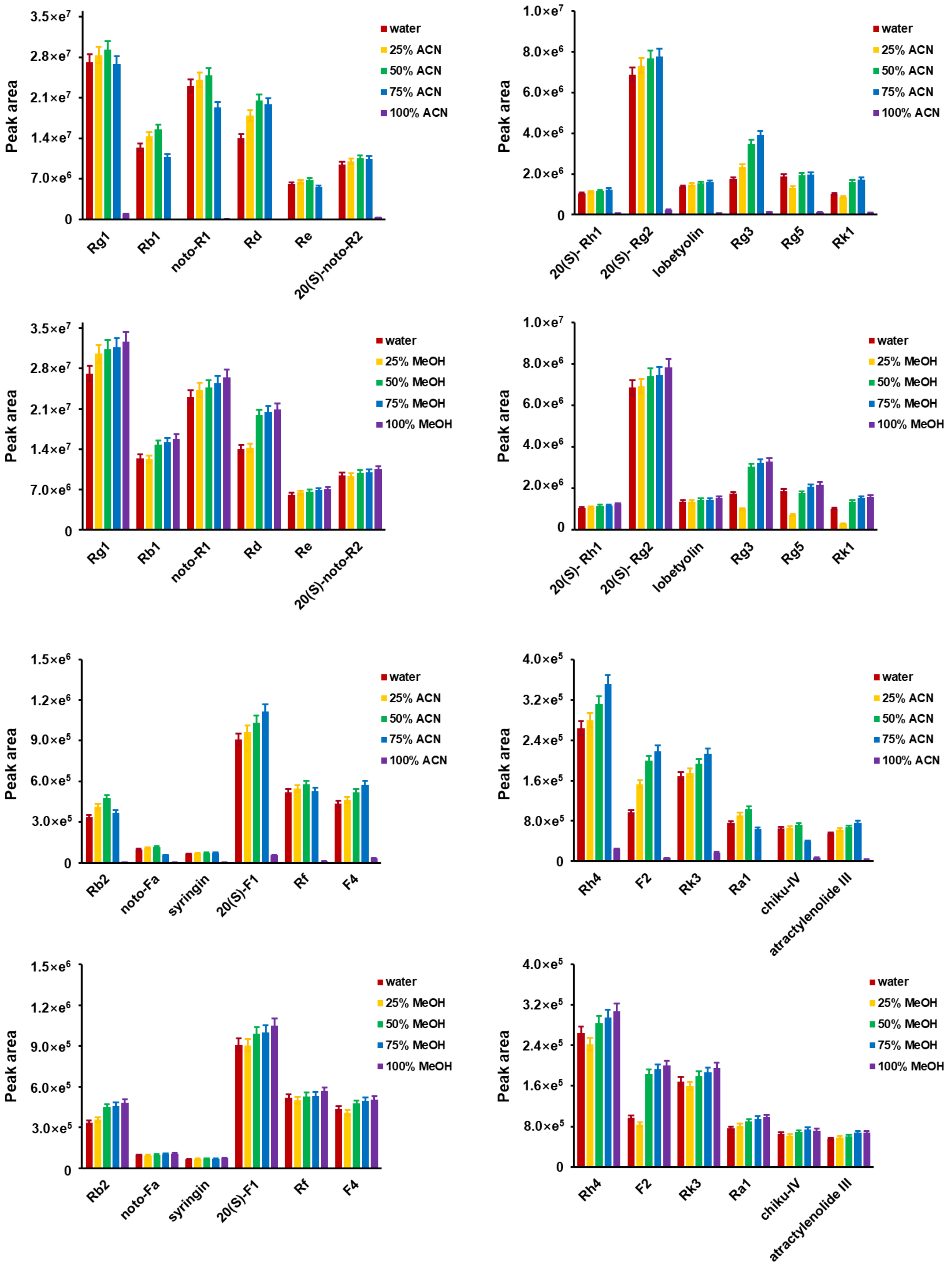

Figure S7. Comparison of different extraction solvents for 27 analytes.

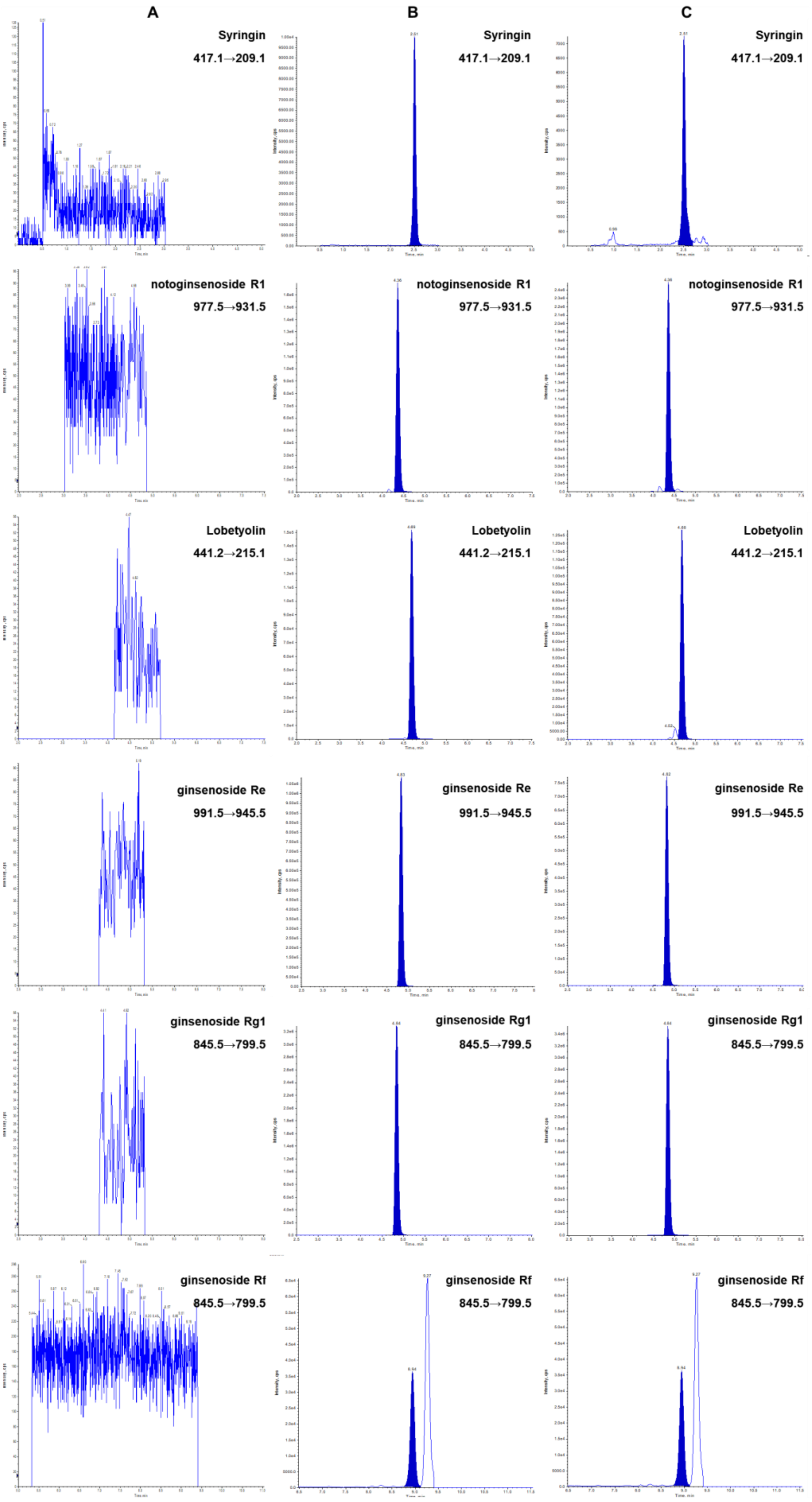

A
B
C
Syringin
417.1→209.1
notoginsenoside R1
977.5→931.5
Lobetyolin
441.2→215.1
ginsenoside Re
991.5→945.5
ginsenoside Rg1
845.5→799.5
ginsenoside Rf
845.5→799.5

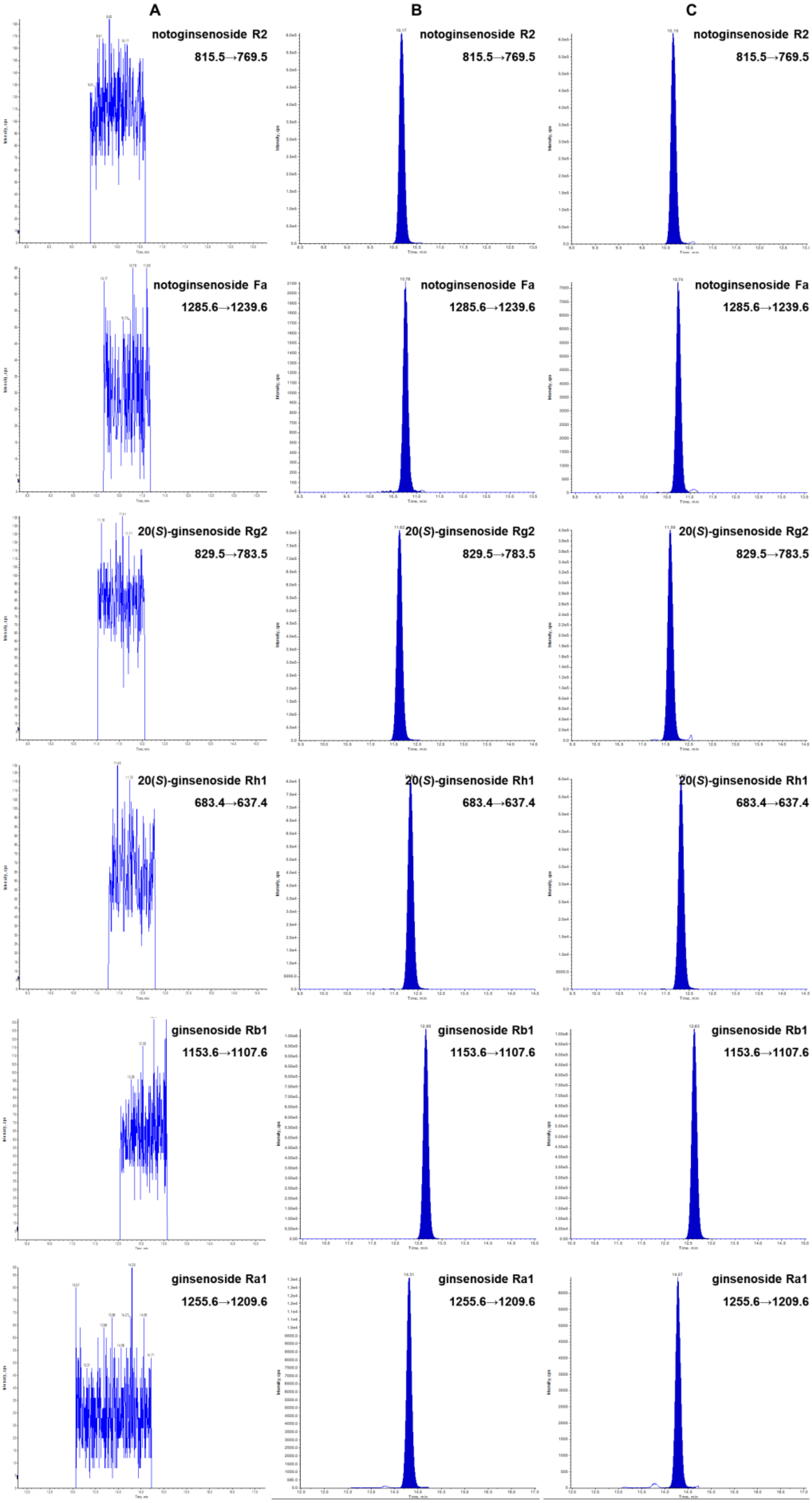
A
B
C
notoginsenoside R2
815.5→769.5
notoginsenoside R2
815.5→769.5
notoginsenoside R2
815.5→769.5
notoginsenoside Fa
1285.6→1239.6
notoginsenoside Fa
1285.6→1239.6
notoginsenoside Fa
1285.6→1239.6
20(S)-ginsenoside Rg2
829.5→783.5
20(S)-ginsenoside Rg2
829.5→783.5
20(S)-ginsenoside Rg2
829.5→783.5
20(S)-ginsenoside Rh1
683.4→637.4
20(S)-ginsenoside Rh1
683.4→637.4
20(S)-ginsenoside Rh1
683.4→637.4
ginsenoside Rb1
1153.6→1107.6
ginsenoside Rb1
1153.6→1107.6
ginsenoside Rb1
1153.6→1107.6
ginsenoside Ra1
1255.6→1209.6
ginsenoside Ra1
1255.6→1209.6
ginsenoside Ra1
1255.6→1209.6

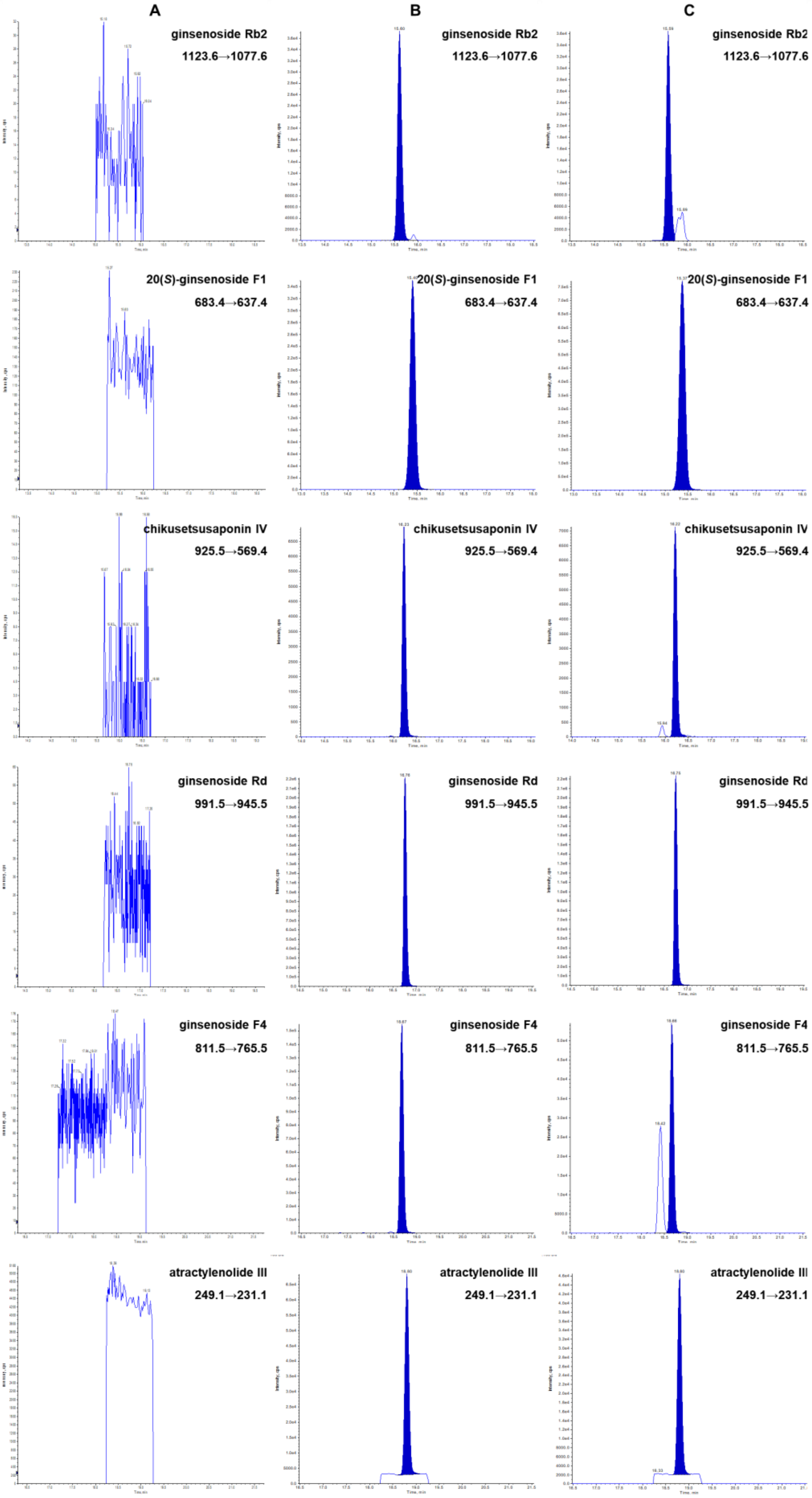
A
B
C
ginsenoside Rb2
1123.6→1077.6
ginsenoside Rb2
1123.6→1077.6
ginsenoside Rb2
1123.6→1077.6
20(S)-ginsenoside F1
683.4→637.4
20(S)-ginsenoside F1
683.4→637.4
20(S)-ginsenoside F1
683.4→637.4
chikusetsusaponin IV
925.5→569.4
chikusetsusaponin IV
925.5→569.4
chikusetsusaponin IV
925.5→569.4
ginsenoside Rd
991.5→945.5
ginsenoside Rd
991.5→945.5
ginsenoside Rd
991.5→945.5
ginsenoside F4
811.5→765.5
ginsenoside F4
811.5→765.5
ginsenoside F4
811.5→765.5
atractylenolide III
249.1→231.1
atractylenolide III
249.1→231.1
atractylenolide III
249.1→231.1

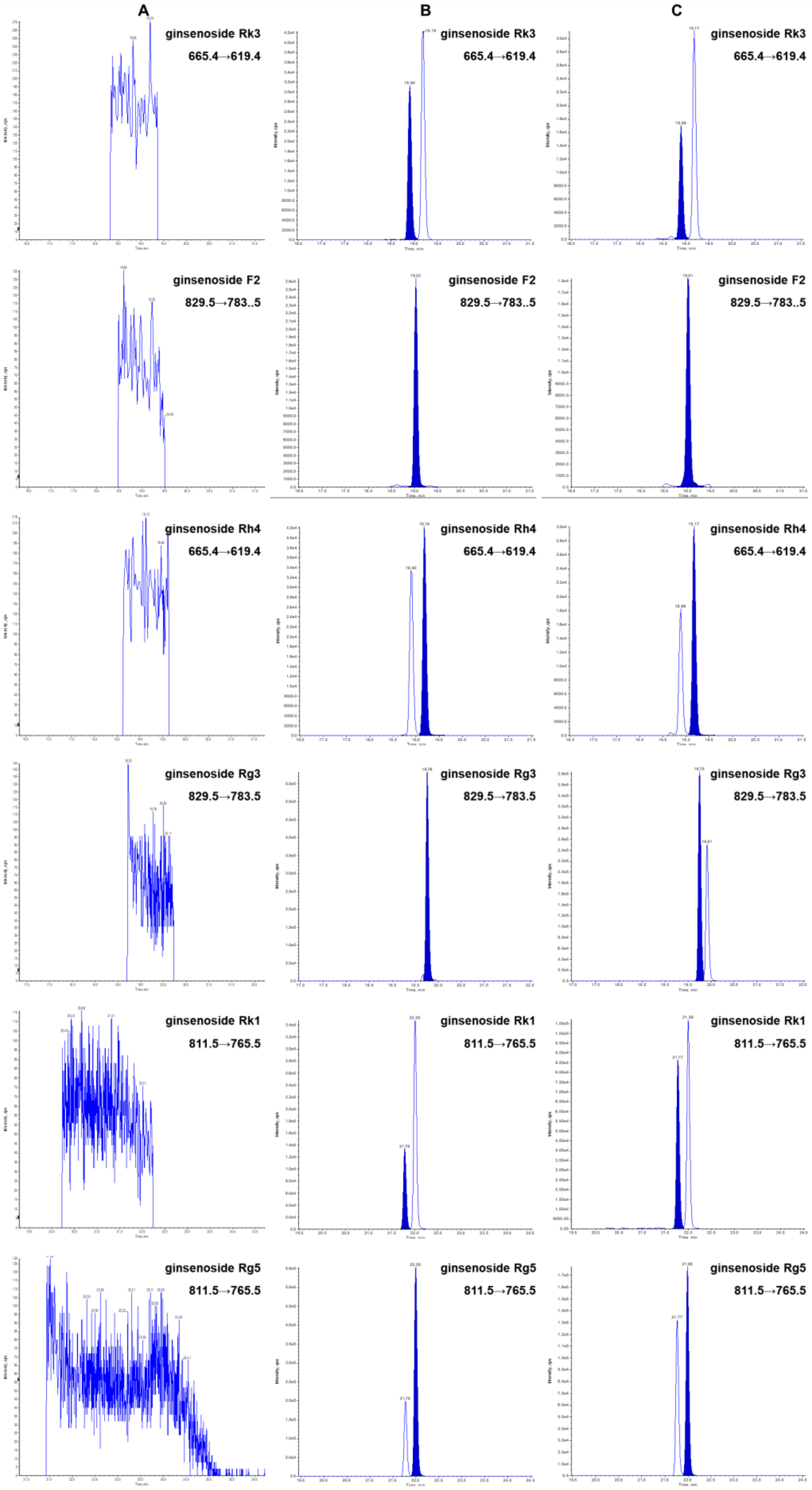

A
B
C
ginsenoside Rk3
665.4→619.4
ginsenoside F2
829.5→783..5
ginsenoside Rh4
665.4→619.4
ginsenoside Rg3
829.5→783.5
ginsenoside Rk1
811.5→765.5
ginsenoside Rg5
811.5→765.5

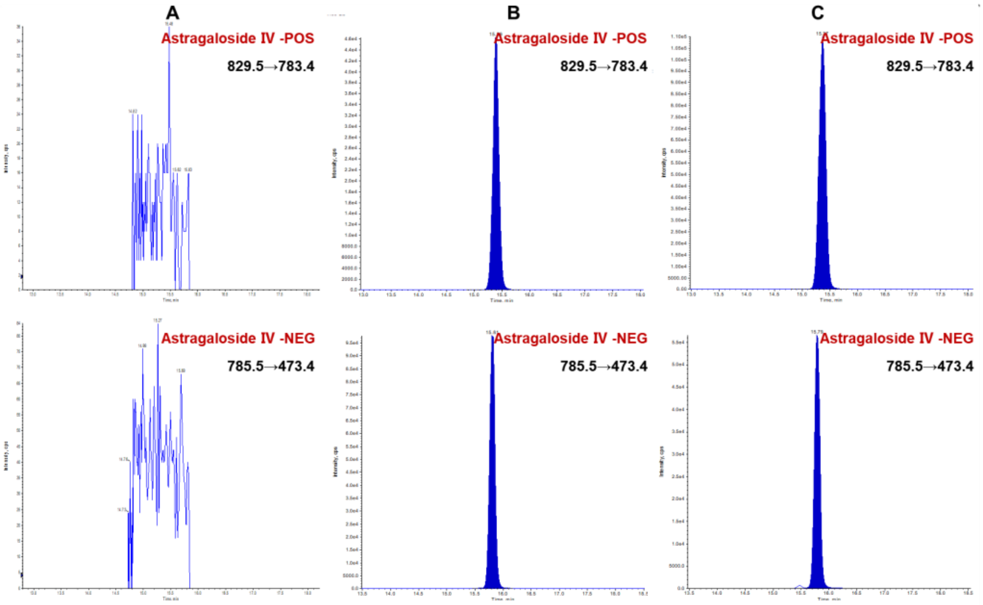

Figure S8. Representative sMRM chromatograms demonstrating the specificity of the established multicomponent quantitative assay approach. (**A**) The blank sample (methanol); (**B**) methanol spiked with the reference compounds and the internal standard; (**C**) the real sample of WXG.

Table S1. Information of 71 reference compounds used in this work.

No.	Compound	Formula	Exact Mass	Subclass
1*	ginsenoside F1	$C_{36}H_{62}O_9$	638.4394	**PPT-type ginsenoside**
2*	ginsenoside Rh1	$C_{36}H_{62}O_9$	638.4394	
3	ginsenoside F3	$C_{41}H_{70}O_{13}$	770.4816	
4*	notoginsenoside R2	$C_{41}H_{70}O_{13}$	770.4816	
5	ginsenoside Rg2	$C_{42}H_{72}O_{13}$	784.4973	
6*	ginsenoside Rf	$C_{42}H_{72}O_{14}$	800.4922	
7*	ginsenoside Rg1	$C_{42}H_{72}O_{14}$	800.4922	
8*	notoginsenoside R1	$C_{47}H_{80}O_{18}$	932.5345	
9*	ginsenoside Re	$C_{48}H_{82}O_{18}$	946.5501	
10	20(*S*)-protopanaxatriol	$C_{30}H_{52}O_4$	476.3866	
11*	20(*R*)-ginsenoside Rg2	$C_{42}H_{72}O_{13}$	784.4973	
12	ginsenoside Rh2	$C_{36}H_{62}O_8$	622.4445	**PPD-type ginsenoside**
13	20(*R*)-ginsenoside Rh2	$C_{36}H_{62}O_8$	622.4445	
14	compound K	$C_{36}H_{62}O_8$	622.4445	
15*	ginsenoside F2	$C_{42}H_{72}O_{13}$	784.4973	
16*	ginsenoside Rg3	$C_{42}H_{72}O_{13}$	784.4973	
17	ginsenoside Rs3	$C_{44}H_{74}O_{14}$	826.5079	
18	notoginsenoside Ft1	$C_{47}H_{80}O_{17}$	916.5396	
19*	ginsenoside Rd	$C_{48}H_{82}O_{18}$	946.5501	
20	malonyl-ginsenoside Rd	$C_{51}H_{84}O_{21}$	1032.5505	
21	notoginsenoside Fd	$C_{47}H_{80}O_{17}$	916.5396	
22	ginsenoside Rd2	$C_{47}H_{80}O_{17}$	916.5396	
23	ginsenoside Rc	$C_{53}H_{90}O_{22}$	1078.5924	
24*	ginsenoside Rb1	$C_{54}H_{92}O_{23}$	1108.6029	
25	malonyl-ginsenoside Rc	$C_{56}H_{92}O_{25}$	1164.5928	
26	malonyl-ginsenoside Rb2	$C_{56}H_{92}O_{25}$	1164.5928	
27	malonyl-ginsenoside Rb1	$C_{57}H_{94}O_{26}$	1194.6033	
28*	ginsenoside Ra1	$C_{58}H_{98}O_{26}$	1210.6346	
29	ginsenoside Ra2	$C_{58}H_{98}O_{26}$	1210.6346	
30	ginsenoside Ra3	$C_{59}H_{100}O_{27}$	1240.6452	
31	notoginsenoside R4	$C_{59}H_{100}O_{27}$	1240.6452	
32*	notoginsenoside Fa	$C_{59}H_{100}O_{28}$	1240.6452	
33	notoginsenoside S	$C_{63}H_{106}O_{30}$	1342.6769	
34*	ginsenoside Rb2	$C_{53}H_{90}O_{22}$	1078.5924	
35	notoginsenoside Fc	$C_{58}H_{98}O_{26}$	1210.6346	
36	ginsenoside Rb3	$C_{53}H_{90}O_{22}$	1078.5924	
37	chikusetsusaponin IVa	$C_{42}H_{66}O_{14}$	794.4453	**OA-type ginsenoside**
38*	chikusetsusaponin IV	$C_{47}H_{74}O_{18}$	926.4875	
39	pseudoginsenoside Rt1	$C_{47}H_{74}O_{18}$	926.4875	
40	ginsenoside Ro	$C_{48}H_{76}O_{19}$	956.4981	
41	24(*R*)-pseudoginsenoside Rt5	$C_{36}H_{62}O_{10}$	654.4343	**OT-type ginsenoside**

42	24(*R*)-pseudoginsenoside F11	$C_{42}H_{72}O_{14}$	800.4922	
43*	ginsenoside Rk3	$C_{36}H_{60}O_{8}$	620.4288	**Other type ginsenoside**
44*	astragaloside IV	$C_{41}H_{68}O_{14}$	784.4609	
45	ginsenoside Rh7	$C_{36}H_{60}O_{9}$	636.4237	
46	ginsenoside Rh8	$C_{36}H_{60}O_{9}$	636.4237	
47*	ginsenoside Rg5	$C_{42}H_{70}O_{12}$	766.4868	
48	ginsenoside Rh3	$C_{36}H_{60}O_{7}$	604.4339	
49	ginsenoside Rg6	$C_{46}H_{70}O_{12}$	766.4867	
50*	ginsenoside F4	$C_{42}H_{70}O_{12}$	766.4867	
51*	ginsenoside Rh4	$C_{36}H_{60}O_{8}$	620.4288	
52*	ginsenoside Rk1	$C_{42}H_{70}O_{12}$	766.4867	
53	ginsenoside Rk2	$C_{36}H_{60}O_{7}$	604.4339	
54	5,6-didehydroginsenoside Rd	$C_{48}H_{80}O_{18}$	944.5345	
55	vinaginsenoside R8	$C_{48}H_{82}O_{19}$	962.5450	
56	gypenoside XLIX	$C_{52}H_{86}O_{21}$	1046.5662	
57	pseudoginsenoside Rh2	$C_{36}H_{62}O_{8}$	622.4445	
58	caffeic acid	$C_{9}H_{8}O_{4}$	180.0423	**Organic acid**
59	vanillic acid	$C_{8}H_{8}O_{4}$	168.0423	
60	isoliquiritigenin	$C_{15}H_{12}O_{4}$	256.0736	**Flavonoid**
61	liquiritin	$C_{21}H_{22}O_{9}$	418.1264	
62	liquiritigenin	$C_{15}H_{12}O_{4}$	256.0736	
63	quercetin	$C_{15}H_{10}O_{7}$	302.0427	
64	kaempferol	$C_{15}H_{10}O_{6}$	286.0477	
65	nardosinone	$C_{15}H_{22}O_{3}$	250.1569	**Other**
66	β-sitosterol	$C_{29}H_{50}O$	414.3862	
67*	lobetyolin	$C_{20}H_{28}O_{8}$	396.1784	
68*	atractylenolide III	$C_{15}H_{20}O_{3}$	248.1412	
69	xanthotoxol	$C_{11}H_{6}O_{4}$	202.0266	
70*	syringin	$C_{17}H_{24}O_{9}$	372.1420	
71	tangshenoside I	$C_{29}H_{42}O_{18}$	678.2371	

* The compounds quantitatively assayed for WXG.

Table S2. Detailed information for the WXG samples analyzed in the current work.

**No.**	**Production batch**	**Specification (g/bag)**	**Sucrose**	**No.**	**Production batch**	**Specification (g/bag)**	**Sucrose**	**No.**	**Production batch**	**Specification (g/bag)**	**Sucrose**
1	1909023	9	T	10	2012027	9	T	19	2012027	5	F
2	2008021	9	T	11	2101037	9	T	20	2012074	5	F
3	2004071	9	T	12	2103003	9	T	21	2009083	5	F
4	2105016	9	T	13	2101038	9	T	22	2012079	5	F
5	2007015	9	T	14	2012028	9	T	23	2105033	5	F
6	2010013	9	T	15	2006008	5	F	24	2012069	5	F
7	2011068	9	T	16	2009018	5	F	25	2104057	5	F
8	2011067	9	T	17	2012030	5	F	26	2104041	5	F
9	2009035	9	T	18	2012029	5	F	27	2105001	5	F

T indicates that the WXG sample contains sucrose; F indicates that the WXG sample is free of sucrose.

Table S3. Detailed information for 20 candidate columns used in stationary phases screening.

No.	Chromatographic column	Specification / Manufacturer	Separation characteristics
1	HSS T3	2.1×100 mm, 1.8 μm; Waters	The universal, silica-based bonded phase used for the HSS T3 sorbents is compatible with 100% aqueous mobile phase and can enhance retention of polar molecules.
2	HSS C18 SB	2.1×100 mm, 1.8 μm; Waters	HSS C18 SB column has the unique non-end-capped, low-coverage silica-based C18 chemistry, which is used for low pH separations that contain complex mixtures of basic and non-basic compounds.
3	CORTECS UPLC C18+	2.1×100 mm, 1.6 μm; Waters	CORTECS C18 columns are general purpose, high-efficiency columns based on a solid-core particle that offer balanced retention of acids, bases and neutrals at low and mid-range pH.
4	BEH Shield RP18	2.1×100 mm, 1.7 μm; Waters	The embedded carbamate group in the bonded phase ligand provides alternate selectivity, especially for phenolic compounds compared to straight chain alkyl columns. This allows for alternate selectivity to that of alkyl reversed-phase columns and aqueous mobile phase compatibility.
5	CSH C18	2.1×100 mm, 1.7 μm; Waters	Based on Ethylene Bridged Hybrid (BEH) particle technology, this column incorporates a low-level surface charge that has been designed to improve sample loadability and peak asymmetry, which is also the first choice for the analysis of peptides.
6	CSH Phenyl-Hexyl	2.1×100 mm, 1.7 μm; Waters	Built on the Charged Surface Hybrid (CSH) particle platform, this column offers exceptional peak shape under both low and high pH conditions while providing complementary selectivity for straight-chain alkyl phases, especially in polyaromatic compounds.
7	HSS Cynao	2.1×100 mm, 1.8 μm; Waters	This chromatographic column has an ultra-performance general purpose propyl cyano bonded phase that could be used for normal- and reversed-phase separations.
8	CSH Fluoro-Phenyl	2.1×100 mm, 1.7 μm; Waters	Designed to provide superior selectivity for positional isomers and polar compounds, the CSH Fluoro-Phenyl column utilizes an intricate combination of multiple retention mechanisms. The non-encapped sorbent can enhance the retention of acidic compounds.
9	ZORBAX Extend C18	2.1×100 mm, 1.8 μm; Agilent	The column incorporates a unique patented bidentate silane, combined with a double-endcapping process that protects the silica from dissolution at high pH, which also has good separations of peptides, polypeptides, and small proteins

			from pH 2–11.5.
10	ZORBAX Eclipse Plus C18	2.1×100 mm, 1.8 μm; Agilent	Filled with high-performance particulate C18 filler, this column could be used for the analysis of acidic and neutral samples, especially for the separation of alkaline compounds with poor peak shape on other chromatographic columns.
11	ZORBAX SB-Aq	2.1×100 mm, 1.8 μm; Agilent	This SB-Aq column has diisopropyl side-chain radical and is compatible with 100% pure water mobile phase, which can be used for high acid mobile phase.
12	Luna Omega Polar C18	2.1×100 mm, 1.6 μm; Phenomenex	Based on silica, this C18 chromatographic column can improve the retention of polar and nonpolar compounds. And its C18 ligand can provide stable hydrophobicity in aqueous solution.
13	Kinetex Biphenyl	2.1×100 mm, 1.7 μm; Phenomenex	With 100% water-soluble solvent stable reversed-phase stationary phase, this biphenyl chromatographic column has hydrophobic selectivity, aromatic selectivity and enhanced polar selectivity.
14	Kinetex XB-C18	2.1×100 mm, 1.7 μm; Phenomenex	As a phenyl shell core column, it is stable in 100% aqueous solution and can provide excellent reverse hydrophobic retention and polar selectivity to aromatic compounds.
15	BEH C18	2.1×100 mm, 1.7 μm; Waters	Built on the BEH particle platform, this column has the widest usable pH range (pH 1–12), which is ideal for the separation of medium or weak polar compounds.
16	CORTECS UPLC Shield RP18	2.1×100 mm, 1.7 μm; Waters	The column utilizes an embedded carbamate group in the bonded phase ligand which provides alternate selectivity, especially for phenolic compounds compared to straight-chain alkyl columns.
17	BEH C8	2.1×100 mm, 1.7 μm; Waters	The trifunctionally bonded BEH particles offer the widest usable pH range (1–12), superior low pH stability, and ultra-low column bleed.
18	Atlantis Premier BEH C18 AX	2.1×100 mm, 1.7 μm; Waters	BEH C18 AX columns provide excellent retention for polar acidic analytes under reversed phase conditions and the reversed phase/anion-exchange mixed mode chemistry is stable from pH 2–10.
19	Exsil Pure 100 C18	2.0×100 mm, 1.5 μm; Exmere Ltd	This chromatographic column has ultra-pure silica bonded stationary phase which can provide superior results for difficult bases and chelates.
20	ZORBAX SB-C18	2.1×100 mm, 1.8 μm; Agilent	This SB-C18 column has diisobutyl side-chain radical and can provide the best stability under the condition of low pH mobile phase.

Table S4. Detailed information of the 205 components characterized from the WXG.

No.	Observed t_R (min)	Observed *m/z*	Molecular Formula	Mass error (ppm)	Observed CCS (Å²)	Adducts	ESI-MS^2	Identification	Type	Source
1	0.82	361.1638	$C_{20}H_{24}O_6$	–2.0	178.74	+H	361.1331, 174.0755, 156.0646	lariciresinol or its isomer	other	CP
2	0.97	341.1078	$C_{12}H_{22}O_{11}$	–3.2	270.32	–H	341.1076, 179.0548, 161.0441, 143.0337, 131.03455, 101.0235	D-(+)-trehalose or its isomer	other	NJ
3	1.39	341.1078	$C_{12}H_{22}O_{11}$	–3.3	255.79	–H	323.0976, 179.0548, 161.0444, 143.0337, 113.0234, 101.0233, 89.0235	D-(+)-trehalose or its isomer	other	NJ
4	2.46	270.1325	$C_{13}H_{19}NO_5$	–4.1	157.61	+H	270.1327, 252.1210, 209.0923, 177.0541, 137.0400	codonopsinol A or its isomer	alkaloid	CP
5	2.73	284.1481	$C_{14}H_{21}NO_5$	–4.1	161.76	+H	284.1479, 266.1376, 238.1226, 180.1011, 88.0735	codonopsinol or its isomer	alkaloid	CP
6	2.81	268.1029	$C_{10}H_{13}N_5O_4$	–4.3	153.53	+H	268.1041, 239.1020, 136.0602, 119.0336	hypoxanthine or its isomer	alkaloid	CP
7	4.73	254.1375	$C_{13}H_{19}NO_4$	–4.7	153.87	+H	254.1376, 161.0584, 150.0764	codonopsinol B or its isomer	alkaloid	CP
8	4.75	416.1908	$C_{19}H_{29}NO_9$	–1.8	192.17	+H	416.1907, 254.1373, 236.1214, 161.0584	codonopiloside A or its isomer	alkaloid	CP
9	4.97	268.1532	$C_{14}H_{21}NO_4$	–4.3	157.29	+H	268.1535, 220.1335, 212.1020, 161.0587, 88.0737	codonopsine or its isomer	alkaloid	CP
10	5.28	416.1907	$C_{19}H_{29}NO_9$	–2.1	195.63	+H	254.1419	codonopiloside A or its isomer	alkaloid	CP
11	6.06	353.0866	$C_{16}H_{18}O_9$	–3.5	167.95	–H	191.0541, 179.0335, 135.0437	chlorogenic acid or its isomer	organic acid	NJ
12[a]	7.86	417.1401	$C_{17}H_{24}O_9$	–0.3	202.20	+HCOO	381.1753	Syringin	other	CP
13	8.13	353.0870	$C_{16}H_{18}O_9$	–2.4	168.52	–H	191.0540, 179.0343, 173.0450, 135.0444, 93.0332	neochlorogenic acid or its isomer	organic acid	PS

14	9.64	287.1378	$C_{16}H_{18}N_2O_3$	–4.3	163.42	+H	287.1376, 269.1272, 251.1176, 233.1052, 209.1071, 207.0901, 193.0757, 182.0826	5-(9H-*β*-carbolin-l-yl)-pentane-1,2,5-triol or its isomer	alkaloid	PS
16	11.94	535.1824	$C_{26}H_{32}O_{12}$	0.5	246.84	–H	373.1285, 343.1174	8-hydroxypinoresinol-4'-*O*-*β*-D-glucopyranoside or its isomer	other	PS
15	11.94	697.2345	$C_{32}H_{42}O_{17}$	–0.6	249.33	–H	373.1285, 343.1174	8-hydroxypinoresinol-4-*O*-(*β*-D-glucopyranosyl)-4'-*O*-*β*-D-glucopyranoside or its isomer	other	PS
17	12.46	325.0916	$C_{15}H_{18}O_8$	–3.8	169.35	–H	119.0494, 101.0376	(*Z*)-2-(b-glucopyranosyloxy)-3-phenylpropenoic acid or its isomer	other	CP
18	12.90	357.1331	$C_{20}H_{22}O_6$	–3.4	246.96	–H	151.0391, 136.0155	(+)-pinoresinol or its isomer	terpenoid	PS
19	12.90	519.1868	$C_{26}H_{32}O_{11}$	–0.8	246.78	–H	357.1332, 151.0391, 136.0155	pinoresinol-4-*O*-*β*-D-glucopyranoside or its isomer	terpenoid	PS
20	12.90	681.2389	$C_{32}H_{42}O_{16}$	–1.7	248.94	–H	357.1332, 151.0391, 136.0155	(+)-pinoresinol *O*-*β*-D-glucopyranosyl-(1→6)-*β*-D-glucopyranoside or its isomer	terpenoid	PS
21	13.54	303.0485	$C_{15}H_{10}O_7$	–4.8	160.53	+H	303.0487, 229.0507	quercetin or its isomer	flavonoid	CP
22	13.60	425.2018	$C_{18}H_{34}O_{11}$	–2.5	209.20	–H	263.1493	hexyl-*β*-gentiobioside or its isomer	other	CP
23	13.79	609.1456	$C_{27}H_{30}O_{16}$	–0.9	228.64	–H	285.0307	rutin hydrate or its isomer	flavonoid	PN
24	13.80	359.1337	$C_{16}H_{24}O_9$	–2.9	180.10	–H	197.0808, 153.0904, 135.0806	7-deoxy-8-epi-loganic acid or its isomer	organic acid	NJ
25	13.92	579.2097	$C_{28}H_{36}O_{13}$	2.4	257.62	–H	417.1539	(+)-syringaresinol-*O*-*β*-D-glucopyranoside or its isomer	terpenoid	PS
26	14.07	535.1813	$C_{26}H_{32}O_{12}$	–1.4	224.44	–H	343.1181	8-hydroxypinoresinol-4'-*O*-*β*-D-glucopyranoside or its isomer	other	NJ

27	14.10	350.1955	$C_{19}H_{28}NO_5$	–2.0	185.10	–e	350.1955, 250.1432, 161.0583, 121.0632	codonopyrrolidium A or its isomer	alkaloid	CP
28	14.20	425.2014	$C_{18}H_{34}O_{11}$	–3.3	202.56	–H	425.2003, 263.1467, 161.0430, 113.0222, 101.0245	hexyl-*β*-gentiobioside or its isomer	other	CP
29	14.28	469.1342	$C_{21}H_{26}O_{12}$	–2.1	203.24	–H	265.0731, 163.0389, 161.0623	tangshenoside V or its isomer	other	CP
30	14.36	535.1828	$C_{26}H_{32}O_{12}$	1.3	225.35	–H	343.1156	8-hydroxypinoresinol-4'-*O*-*β*-D-glucopyranoside or its isomer	other	NJ
31	14.93	521.2006	$C_{26}H_{34}O_{11}$	–4.3	224.26	–H	329.1349	isolariciresinol 9′-*O*-*β*-D-glucopyranoside or its isomer	terpenoid	PS
32	15.04	352.2110	$C_{19}H_{30}NO_5$	–2.4	182.83	–e	352.2101, 250.1412, 220.1319, 205.0843, 161.0580, 88.0733	codotubulosine B or its isomer	alkaloid	CP
33	15.26	352.2109	$C_{19}H_{30}NO_5$	–2.7	185.86	–e	352.2112, 250.1437, 220.1316, 205.0836, 161.0583, 88.0738	codotubulosine B or its isomer	alkaloid	CP
34	15.48	609.1454	$C_{27}H_{30}O_{16}$	–1.1	229.35	–H	609.1438, 315.0485, 314.0416, 300.0253, 299.0172, 285.0363, 271.0235	rutin hydrate or its isomer	flavonoid	PN
35	16.32	515.1180	$C_{25}H_{24}O_{12}$	–2.9	212.29	–H	353.0860, 191.0524, 179.0318, 173.0444	1,5-di-*O*-caffeoylquinic acid or its isomer	organic acid	NJ
36	16.61	519.1866	$C_{26}H_{32}O_{11}$	–1.1	224.14	–H	357.1321	pinoresinol-4-*O*-*β*-D-glucopyranoside or its isomer	terpenoid	NJ
37	16.62	357.1328	$C_{20}H_{22}O_6$	–4.3	202.72	–H	136.0143	(+)-pinoresinol or its isomer	terpenoid	PS
38	17.92	1093.5808	$C_{53}H_{90}O_{23}$	0.7	328.13	–H	1093.5790, 961.5406	chikusetsusaponin LM5 or its isomer	ginsenoside	PN
40	17.99	515.1195	$C_{25}H_{24}O_{12}$	0.1	207.07	–H	353.0865, 179.0323, 135.0432	1,5-di-*O*-caffeoylquinic acid or its isomer	organic acid	NJ

39	17.99	753.4072	$C_{39}H_{60}O_{14}$	2.2	298.17	+H	753.4084, 591.3538, 573.3427, 429.2994	ophiopogonin C′ or its isomer	steroidal saponin	PS
42	18.00	753.4079	$C_{39}H_{60}O_{14}$	3.1	296.04	+H	753.4110, 591.3520, 573.3420, 297.2173	ophiopogonin C′ or its isomer	steroidal saponin	PS
41	18.00	1077.5137	$C_{51}H_{80}O_{24}$	2.3	313.91	+H	1077.5126, 753.4084, 591.3538, 573.3427, 429.2994	(25R)-spirost-5-en-12-one-3-*O*-*β*-D-glucopyranosyl-(1→2)-*β*- D-glucopyranosyl-(1→3)-*β*-D-glucopyranosyl-(1→4)-*β*-D-galactopyranoside or its isomer	steroidal saponin	PS
43	18.38	1139.5880	$C_{53}H_{90}O_{23}$	2.2	336.00	+HCOO	1093.5832, 961.5382	chikusetsusaponin LM5 or its isomer	ginsenoside	PN
44	18.39	373.1289	$C_{20}H_{22}O_{7}$	–1.1	190.23	–H	313.1036, 269.0784	(+)-1-hydroxypinoresinol or its isomer	other	NJ
45	18.41	1225.5495	$C_{56}H_{90}O_{29}$	0.0	350.19	–H	1225.5490, 1093.5832, 961.5382	chikusetsusaponin LM6 or its isomer	ginsenoside	PN
46	18.48	753.4079	$C_{39}H_{60}O_{14}$	3.1	297.83	+H	753.4089, 591.3530, 429.2987	ophiopogonin C′ or its isomer	steroidal saponin	PS
47	18.49	1047.5031	$C_{50}H_{78}O_{23}$	2.3	313.35	+H	1047.5060, 753.4089, 591.3530, 429.2987	spirost-5-en-12-one-3-*O*-*β*-D-glucopyranosyl-(1→2)-[*β*-D-xylopyranosyl-(1→3)]-*β*-D-glucopyranosyl-(1→4)-*β*-D-galactopyranoside or its isomer	steroidal saponin	PS
49	18.50	753.4073	$C_{39}H_{60}O_{14}$	2.3	297.60	+H	753.4123, 591.3519	ophiopogonin C′ or its isomer	steroidal saponin	PS
48	18.50	1063.4963	$C_{50}H_{80}O_{24}$	–0.3	318.24	–H	1063.4974, 931.4547	sibiricogenin 3-*O*-*β*-lycotetraoside or its isomer	other	NJ

50	18.51	915.4580	$C_{45}H_{70}O_{19}$	–0.5	296.44	+H	753.4089, 591.3530, 429.2987	(25S)-pratioside D1 or its isomer	steroidal saponin	PS
51[a]	18.56	395.1695	$C_{20}H_{28}O_{8}$	–4.0	216.17	–H	484.0590	lobetyolin	other	CP
52	18.66	961.5369	$C_{48}H_{82}O_{19}$	–0.8	312.84	–H	961.5362, 799.4862, 637.4317, 475.3796	floralginsenoside La (24α) or its isomer	ginsenoside	PN
54	18.70	753.4068	$C_{39}H_{60}O_{14}$	1.5	298.57	+H	753.4074, 591.3537, 429.3001	(25*S*)-kingianoside A or its isomer	steroidal saponin	PS
53	18.70	1047.5021	$C_{50}H_{78}O_{23}$	1.3	309.69	+H	915.4616, 753.4074, 591.3537, 429.3001	spirost-5-en-12-one-3-*O*-*β*-D-glucopyranosyl-(1→2)-[*β*-D-xylopyranosyl-(1→3)]-*β*-D-glucopyranosyl-(1→4)-*β*-D-galactopyranoside or its isomer	steroidal saponin	PS
55	18.71	1063.4965	$C_{50}H_{80}O_{24}$	–0.2	316.80	–H	931.4524, 799.4862, 637.4317, 475.3796	PPT-2Glc-2Xyl or its isomer	ginsenoside	PN
56	18.73	915.4604	$C_{45}H_{70}O_{19}$	2.2	294.56	+H	915.4616, 753.4074, 591.3537, 429.3001	(25*S*)-pratioside D1 or its isomer	steroidal saponin	PS
57	18.74	931.4532	$C_{45}H_{72}O_{20}$	–1.3	301.83	–H	931.4524, 769.4029, 751.3830	kingianoside C or its isomer	steroidal saponin	PS
58	18.79	1139.5855	$C_{53}H_{90}O_{23}$	0.0	339.35	+HCOO	1093.5798, 931.5321	floralginsenoside P or its isomer	ginsenoside	PN
61	18.89	753.4075	$C_{39}H_{60}O_{14}$	2.5	298.70	+H	753.4087, 591.3524, 429.2997	(25*S*)-kingianoside A or its isomer	steroidal saponin	PS
62	18.89	1047.5054	$C_{50}H_{78}O_{23}$	4.5	307.22	+H	1047.5086, 753.4087, 591.3524, 429.2997	spirost-5-en-12-one-3-*O*-*β*-D-glucopyranosyl-(1→2)-[*β*-D-xylopyranosyl-(1→3)]-*β*-D-glucopyranosyl-(1→4)-*β*-D-galactopyranoside or its isomer	steroidal saponin	PS

60	18.89	1063.4989	$C_{50}H_{80}O_{24}$	2.1	318.55	–H	1063.4979, 931.4534	sibiricogenin 3-*O*-*β*-lycotetraoside or its isomer	other	NJ
59	18.89	1225.5507	$C_{56}H_{90}O_{29}$	1.0	354.55	–H	1063.4979, 931.4534	chikusetsusaponin LM6 or its isomer	ginsenoside	PN
63	18.91	1063.4982	$C_{50}H_{80}O_{24}$	1.4	318.23	–H	1063.4984, 931.4565	sibiricogenin 3-*O*-*β*-lycotetraoside or its isomer	other	NJ
64	18.96	235.1329	$C_{14}H_{20}O_{3}$	–4.6	158.18	–H	191.1440	tetradeca-4*E*,8*E*,12*E*-triene-10-yne-1,6,7-triol or its isomer	other	CP
65	19.12	833.4916	$C_{41}H_{72}O_{14}$	1.4	282.12	+HCOO	787.4845, 493.3900	OT+2H-Xyl-Glc	ginsenoside	PN
66	19.49	831.4738	$C_{41}H_{70}O_{14}$	–1.1	283.45	+HCOO	785.4710, 653.4335, 491.3745	pseudoginsenoside Rt2 (24*R*) or its isomer	ginsenoside	PN
67	19.67	284.1268	$C_{17}H_{17}NO_{3}$	–4.7	169.42	+H	147.0428, 119.0486	N-trans-p-coumaroyltyramine or its isomer	alkaloid	PS
68	19.76	961.5371	$C_{48}H_{82}O_{19}$	–0.7	319.34	–H	961.5365, 799.4846, 637.4315, 475.3783	ginsenoside Re1 or its isomer	ginsenoside	PN
69	19.77	769.4022	$C_{39}H_{62}O_{15}$	0.8	276.22	–H	769.4029, 637.4315, 475.3783	chikusetsusaponin LM1 or its isomer	ginsenoside	PN
70	19.79	931.4539	$C_{45}H_{72}O_{20}$	–0.5	296.29	–H	799.4846, 637.4315, 475.3783	kingianoside C or its isomer	steroidal saponin	PS
71	19.92	755.4222	$C_{39}H_{62}O_{14}$	1.3	277.81	+H	755.4196, 593.3722, 269.1897	huangjinoside E or its isomer	steroidal saponin	PS
72[a]	20.39	977.5326	$C_{47}H_{80}O_{18}$	–0.1	320.21	+HCOO	931.5261, 799.4834, 637.4312, 475.3772	notoginsenoside R1	ginsenoside	PN
73	20.68	314.1379	$C_{18}H_{19}NO_{4}$	–2.5	179.07	+H	314.2197, 177.0538, 145.0273	3-(4-hydroxy)-N-[2-(4-hydroxyphenyl)-2-methoxyethyl]-2-propenamide or its isomer	alkaloid	PS

74	20.88	977.5321	$C_{47}H_{80}O_{18}$	–0.6	316.44	+HCOO	931.5254, 799.4847, 637.4319, 475.3780	notoginsenoside Fp1 or its isomer	ginsenoside	PN
75	21.42	637.4326	$C_{36}H_{62}O_{9}$	0.8	265.71	–H	637.4316, 475.3787, 391.2841, 179.0543, 161.0441	3-*O*-*β*-D-glucopyranosyl-20(*S*)-protopanaxatriol or its isomer	ginsenoside	PN
76[a]	21.42	799.4854	$C_{42}H_{72}O_{14}$	0.6	288.21	–H	845.4896, 799.4843, 637.4316, 475.3887, 391.2841, 161.0441	ginsenoside Rg1	ginsenoside	PN
77[a]	21.50	945.5431	$C_{48}H_{82}O_{18}$	0.3	321.25	–H	945.5421, 783.4895, 621.4306, 457.3688, 161.0441	ginsenoside Re	ginsenoside	PN
78	21.86	461.2018	$C_{21}H_{34}O_{11}$	–2.2	206.03	–H	461.2034, 347.1719	urceolide or its isomer	other	NJ
79	21.88	309.0859	$C_{17}H_{12}N_{2}O_{4}$	–3.4	166.01	+H	291.0760, 263.0802, 235.0853, 206.0823, 180.0797	flazine or its isomer	alkaloid	PS
80	22.13	285.0747	$C_{16}H_{12}O_{5}$	–3.7	158.14	+H	283.0614, 268.0357	acacetin or its isomer	flavonoid	PS
81	22.17	591.1729	$C_{28}H_{32}O_{14}$	1.7	240.08	–H	283.0614, 268.0357	acaciin, acacetin-7-O-rutinosid, buddleoside, linarine or its isomer	flavonoid	PS
82	22.17	931.4538	$C_{45}H_{72}O_{20}$	–0.7	287.93	–H	931.5249, 799.4834, 637.4342,	kingianoside C or its isomer	steroidal saponin	PS
83	22.57	841.4959	$C_{44}H_{74}O_{15}$	0.5	293.34	–H	841.4942, 799.4855, 781.4729, 637.4343, 619.4196, 475.3790	6'-*O*-acetyl-ginsenoside Rg1 or its isomer	ginsenoside	PN
84	22.60	1123.5921	$C_{54}H_{92}O_{24}$	1.3	220.65	–H	1123.5905, 961.5432, 799.4855, 637.4343, 619.4196, 475.3790	6-*O*-[*β*-D-glucopyranosyl-(1→2)-*β*-D-glucopyranosyl]-20-*O*-[*β*-D-glucopyranosyl-(1→4)-*β*-D-glucopyranosyl]-20(*S*)-protopanaxatriol or its isomer	ginsenoside	PN
85	22.85	979.5511	$C_{48}H_{84}O_{20}$	2.9	202.70	–H	979.5488, 799.4864, 475.3806	vinaginsenoside R13 or its isomer	ginsenoside	PN
86	22.88	947.5236	$C_{46}H_{78}O_{17}$	1.6	312.16	+HCOO	901.5166, 769.4731, 607.4235, 475.3754	chikusetsusaponin LM2 or its isomer	ginsenoside	PN

88	23.05	577.3738	$C_{33}H_{52}O_{8}$	0.6	248.48	+H	577.3738, 415.3213, 253.1953	trillin or its isomer	steroidal saponin	PS
87	23.05	739.4276	$C_{39}H_{62}O_{13}$	1.7	298.59	+H	739.4288, 591.3542, 577.3738, 415.3213, 253.1953	funkioside C or its isomer	steroidal saponin	PS
89	23.26	753.4072	$C_{39}H_{60}O_{14}$	2.2	301.42	+H	591.3533	ophiopogonin C′ or its isomer	steroidal saponin	PS
90	23.28	1075.4961	$C_{51}H_{80}O_{24}$	–0.5	310.38	–H	1075.4963, 913.4403	(25*R*)-spirost-5-en-3*β*,17*α*-diol-3-*O*-*α*-L-rhamnopyranosyl-(1→4)-*α*-L-rhamnopyranosyl-(1→4)-[α-L-rhamnopyranosyl-(1→2)]-*β*-D-glucopyranoside or its isomer	steroidal saponin	PS
92	23.30	739.4286	$C_{39}H_{62}O_{13}$	3.0	300.14	+H	739.4256, 591.3532, 577.3753, 253.1950	funkioside C or its isomer	steroidal saponin	PS
91	23.30	947.5236	$C_{46}H_{78}O_{17}$	1.6	315.06	+HCOO	901.5136, 637.4331, 755.4212, 475.3819	chikusetsusaponin L5 or its isomer	ginsenoside	PN
93	23.54	841.4951	$C_{44}H_{74}O_{15}$	–0.4	298.47	–H	783.3796, 637.4317, 619.4212, 475.3807	6'-*O*-acetyl-ginsenoside Rg1 or its isomer	ginsenoside	PN
94	23.59	815.4784	$C_{41}H_{70}O_{13}$	–1.8	290.08	+HCOO	769.4753, 607.4209, 475.3807	chikusetsusaponin LM1 or its isomer	ginsenoside	PN
95	23.61	285.0754	$C_{16}H_{12}O_{5}$	–1.2	203.15	+H	285.0752, 270.0516	acacetin or its isomer	flavonoid	PS
96	23.62	739.4270	$C_{39}H_{62}O_{13}$	0.9	300.51	+H	739.4283, 577.3741	funkioside C or its isomer	steroidal saponin	PS
97	23.63	283.0601	$C_{16}H_{12}O_{5}$	–3.8	206.23	–H	268.0365	acacetin or its isomer	flavonoid	PS
98	23.64	959.5208	$C_{48}H_{80}O_{19}$	–1.4	326.86	–H	959.5202, 797.4682, 635.4166, 473.3633	ginsenoside III or its isomer	ginsenoside	PN

99	23.64	1033.5232	$C_{50}H_{80}O_{22}$	1.7	317.11	+H	1033.5253, 739.4283, 577.3741	spirost-5-en-3β,14α-diol-3-*O*-*β*-*D*-glucopyranosyl-(1→2)-[*β*-D-xylopyranosyl-(1→3)]-*β*-*D*-glucopyranosyl-(1→4)- *β*-*D*-galactopyranoside or its isomer	steroidal saponin	PS
101	23.83	871.4720	$C_{44}H_{70}O_{17}$	4.0	339.80	+H	871.4724, 709.4168, 577.3743, 415.3205, 253.1945	polygonatoside C1 or its isomer	steroidal saponin	PS
100	23.83	885.4859	$C_{45}H_{72}O_{17}$	1.9	309.55	+H	885.4870, 739.4279, 577.3743, 415.3205, 253.1945	gracillin or its isomer	steroidal saponin	PS
102	23.84	739.4283	$C_{39}H_{62}O_{13}$	2.7	299.20	+H	739.4279, 577.3743, 415.3205, 253.1945	funkioside C or its isomer	steroidal saponin	PS
103	23.84	1163.5885	$C_{56}H_{90}O_{25}$	3.5	342.10	+H	1163.5879, 1001.5364, 869.4925, 723.4334, 577.3745, 415.3195, 253.1966	$C_{27}H_{42}O_3$-2Glc-2Rha-Xyl	ginsenoside	PN
104	23.85	577.3744	$C_{33}H_{52}O_8$	1.5	247.73	+H	577.3743, 415.3205, 253.1945	trillin or its isomer	steroidal saponin	PS
105	23.94	901.4825	$C_{45}H_{72}O_{18}$	3.7	295.51	+H	901.4824, 739.4289, 577.3744, 415.3210, 397.3092, 271.2052, 253.1940	neosibiricoside D or its isomer	steroidal saponin	PS
106	24.02	1031.5080	$C_{50}H_{80}O_{22}$	1.1	330.71	–H	1031.5066, 899.4628, 637.4302	spirost-5-en-3*β*,14α-diol-3-*O*-*β*-D-glucopyranosyl-(1→2)-[*β*-*D*-xylopyranosyl-(1→3)]-*β*-D-glucopyranosyl-(1→4)-*β*-D-galactopyranoside or its isomer	steroidal saponin	PS
107	24.39	1031.5451	$C_{51}H_{82}O_{21}$	2.9	356.60	+H	1031.5429, 869.4905, 723.4392, 577.3690, 415.3200, 253.1938	$C_{27}H_{42}O_3$-2Glc-2Rha	ginsenoside	PN

108	24.41	961.5380	$C_{47}H_{80}O_{17}$	0.2	318.64	+HCOO	915.5312, 783.4898, 475.3771	vinaginsenoside R18 or its isomer	ginsenoside	PN
111	24.49	577.3740	$C_{33}H_{52}O_8$	0.8	249.99	+H	577.3739, 415.3201, 253.1943	trillin or its isomer	steroidal saponin	PS
110	24.49	739.4285	$C_{39}H_{62}O_{13}$	3.0	301.43	+H	739.4282, 577.3739, 415.3201, 253.1943	funkioside C or its isomer	steroidal saponin	PS
109	24.49	871.4715	$C_{44}H_{70}O_{17}$	3.4	295.99	+H	871.4704, 739.4282, 577.3739, 415.3201, 253.1943, 157.1006	polygonatoside C1 or its isomer	steroidal saponin	PS
112	24.53	1017.5304	$C_{50}H_{80}O_{21}$	3.8	353.14	+H	1017.5295, 855.4759, 723.4309, 577.3751, 415.3201, 271.2054, 253.1938	$C_{27}H_{42}O_3$-2Glc-Rha-Xyl	ginsenoside	PN
113	24.55	815.4792	$C_{41}H_{70}O_{13}$	–0.8	293.64	+HCOO	815.4796, 769.4745, 637.4321, 475.3795	chikusetsusaponin LM1 or its isomer	ginsenoside	PN
114	24.83	885.4866	$C_{45}H_{72}O_{17}$	2.7	314.49	+H	885.4867, 723.4326, 579.3170, 577.3739, 415.3180, 271.2049, 253.1950	gracillin or its isomer	steroidal saponin	PS
115	24.93	1047.5388	$C_{51}H_{82}O_{22}$	1.7	316.98	+H	885.4860, 739.4276, 723.4323, 577.3735, 415.3200, 253.1941	parissaponin Pb or its isomer	steroidal saponin	PS
117	24.96	577.3737	$C_{33}H_{52}O_8$	0.4	247.81	+H	577.3535, 415.3200, 253.1941	trillin or its isomer	steroidal saponin	PS
116	24.96	739.4278	$C_{39}H_{62}O_{13}$	2.0	298.80	+H	739.4276, 577.3735, 415.3200, 253.1941	funkioside C or its isomer	steroidal saponin	PS
118	24.97	885.4865	$C_{45}H_{72}O_{17}$	2.6	311.31	+H	885.4860, 723.4323, 577.3735, 415.3200, 253.1941	gracillin or its isomer	steroidal saponin	PS
119	25.70	961.5385	$C_{48}H_{82}O_{19}$	0.8	328.11	–H	961.5367, 799.4842, 637.4323, 475.3800	notoginsenoside R3 or its isomer	ginsenoside	PN

120	25.79	219.1735	$C_{15}H_{22}O$	–3.9	151.26	+H	219.1731, 203.1422	(-)-(14β,15β)-aristolone or its isomer	other	NJ
121	26.14	827.4786	$C_{42}H_{70}O_{13}$	–1.5	303.18	+HCOO	827.4760, 781.4737, 619.4212	ginsenoside Rh15 or its isomer	ginsenoside	PN
122	26.17	913.4429	$C_{45}H_{70}O_{19}$	–1.0	293.92	+HCOO	867.4353, 721.3801	(*25S*)-pratioside D1 or its isomer	steroidal saponin	PS
123	26.25	929.4366	$C_{45}H_{70}O_{20}$	–2.3	299.05	–H	929.4389, 767.3845	vinaginsenoside R3 or its isomer	ginsenoside	PN
124	26.65	931.4539	$C_{45}H_{72}O_{20}$	–0.6	304.96	–H	931.4533, 769.3995	kingianoside C or its isomer	steroidal saponin	PS
125	26.85	327.2162	$C_{18}H_{32}O_5$	–4.7	180.26	–H	327.2196, 229.1445, 211.1330, 171.1018	9,12,13-trihydroxy-10,15-octadecadienoic acid or its isomer	organic acid	CP
126	26.92	869.4540	$C_{44}H_{70}O_{17}$	0.0	290.94	–H	869.4546, 723.3958	polygonatoside C1 or its isomer	steroidal saponin	PS
128	27.21	769.4024	$C_{39}H_{62}O_{15}$	1.1	273.07	–H	637.4320, 475.3798, 391.2860	chikusetsusaponin LM1 or its isomer	ginsenoside	PN
127	27.21	1077.5126	$C_{51}H_{82}O_{24}$	0.3	366.80	–H	799.4849, 637.4320, 475.3798, 391.2860	floralginsenoside M or its isomer	ginsenoside	PN
129[a]	27.23	799.4854	$C_{42}H_{72}O_{14}$	0.5	303.56	–H	799.4849, 637.4320, 475.3798, 391.2860	ginsenoside Rf	ginsenoside	PN
130	27.49	1371.6807	$C_{64}H_{108}O_{31}$	0.4	391.70	–H	1371.6809, 1239.6395, 1107.5988, 945.5421, 783.4893, 619,3137	notoginsenoside T or its isomer	ginsenoside	PN
131	27.81	913.5164	$C_{46}H_{76}O_{15}$	–0.3	299.90	+HCOO	913.5251, 867.5154, 799.4831, 781.4712, 637.4236	koryoginsenoside R1 or its isomer	ginsenoside	PN
132	28.37	799.4847	$C_{42}H_{72}O_{14}$	–0.2	295.98	–H	799.4838, 475.3790	notoginsenoside U or its isomer	ginsenoside	PN
134[a]	28.71	815.4796	$C_{41}H_{70}O_{13}$	–0.3	296.60	+HCOO	769.4738, 637.4315, 475.3785, 391.2843, 161.0439	notoginsenoside R2	ginsenoside	PN

133	28.71	883.4673	$C_{44}H_{70}O_{15}$	–2.7	308.63	+HCOO	769.4738, 637.4315, 475.3785, 391.2843, 161.0439	PPT-Glc-Xyl-But	ginsenoside	PN
135[a]	29.11	1239.6390	$C_{59}H_{100}O_{27}$	0.9	364.21	–H	1239.6375, 1107.5948, 945.5413, 783.4900, 459.3826	notoginsenoside R4	ginsenoside	PN
136	29.19	815.4809	$C_{41}H_{70}O_{13}$	1.3	295.41	+HCOO	769.4738, 637.4332, 475.3802	chikusetsusaponin LM1 or its isomer	ginsenoside	PN
137	29.50	901.5163	$C_{46}H_{78}O_{17}$	–0.4	315.46	–H	901.5150, 769.4733, 475.3780, 191.0548, 149.0439, 131.0332	chikusetsusaponin L5 or its isomer	ginsenoside	PN
138	30.03	329.2323	$C_{18}H_{34}O_5$	–3.1	182.41	–H	329.2325, 229.1431, 211.1332, 183.1356, 171.1022	9,10,13-trihydroxy-(*E*)-11-octadecenoic acid or its isomer	organic acid	CP
139	30.42	769.4741	$C_{41}H_{70}O_{13}$	–0.3	300.76	–H	769.4737, 637.4326, 475.3787, 391.2842	chikusetsusaponin LM1 or its isomer	ginsenoside	PN
140[a]	30.66	783.4897	$C_{42}H_{72}O_{13}$	–0.5	296.14	–H	783.4891, 637.4312, 475.3782, 391.2839, 161.0442	20(*S*)-ginsenoside Rg2	ginsenoside	PN
141	30.72	577.3736	$C_{33}H_{52}O_8$	0.2	249.36	+H	577.3734, 415.3203, 253.1946	trillin or its isomer	steroidal saponin	PS
142[a]	30.91	683.4373	$C_{36}H_{62}O_9$	–0.4	274.41	+HCOO	683.4371, 637.4316, 475.3782, 391.2844, 161.0436	20(*S*)-ginsenoside Rh1	ginsenoside	PN
143[a]	31.18	1239.6379	$C_{59}H_{100}O_{27}$	0.0	368.50	–H	1239.6375, 1107.5948, 945.5413, 783.4900, 621.4374, 459.3826	notoginsenoside Fa	ginsenoside	PN
144	31.48	577.3739	$C_{33}H_{52}O_8$	0.6	248.03	+H	577.3736, 415.3201, 253.1944	trillin or its isomer	steroidal saponin	PS
146	31.48	739.4275	$C_{39}H_{62}O_{13}$	1.7	299.39	+H	739.4282, 577.3736, 415.3201, 271.2053, 253.1944	funkioside C or its isomer	steroidal saponin	PS
145	31.48	871.4712	$C_{44}H_{70}O_{17}$	3.0	341.13	+H	871.4712, 709.4168, 577.3736, 415.3201, 253.1944	polygonatoside C1 or its isomer	steroidal saponin	PS

147	31.48	885.4864	$C_{45}H_{72}O_{17}$	2.5	327.04	+H	739.4282, 723.4317, 577.3736, 415.3201, 253.1944	gracillin or its isomer	steroidal saponin	PS
148	31.5	577.3740	$C_{33}H_{52}O_{8}$	0.8	247.77	+H	415.3220, 271.2037	trillin or its isomer	steroidal saponin	PS
149[a]	31.51	783.4903	$C_{42}H_{72}O_{13}$	0.3	301.07	–H	783.4893, 637.4320, 475.3791	20(*R*)-ginsenoside Rg2	ginsenoside	PN
150	31.84	1105.5804	$C_{54}H_{90}O_{23}$	0.4	345.59	–H	1105.5794, 943.5286, 781.4741	5,6-didehydroginsenoside Rb1 or its isomer	ginsenoside	PN
151	32.51	683.4374	$C_{36}H_{62}O_{9}$	–0.3	279.53	+HCOO	683.4376, 637.4324, 475.3785	chikusetsusaponin L10 or its isomer	ginsenoside	PN
152	33.64	577.3735	$C_{33}H_{52}O_{8}$	0.1	249.06	+H	577.3720, 415.3192, 271.2055, 253.1944	trillin or its isomer	steroidal saponin	PS
153	33.71	739.4276	$C_{39}H_{62}O_{13}$	1.8	300.38	+H	739.4267, 577.3730, 415.3204, 271.2046, 253.1944	funkioside C or its isomer	steroidal saponin	PS
154	33.72	871.4705	$C_{44}H_{70}O_{17}$	2.2	343.14	+H	871.4703, 709.4170, 577.3730, 415.3204, 271.2046, 253.1944	polygonatoside C1 or its isomer	steroidal saponin	PS
155	34.24	203.1787	$C_{15}H_{22}$	–3.6	212.40	+H	203.1785, 189.1628, 173.1321, 133.1001, 119.0841, 105.0683, 95.0841	α-Vatirenene or its isomer	other	NJ
156	34.27	783.4885	$C_{42}H_{72}O_{13}$	–2.0	302.44	–H	783.4902, 765.4792, 621.4374, 459.3838, 221.0655	3-*O*-*β*-D-glucopyranosyl-(1→2)-*β*-D-glucopyranoside-12*β*,25-dihydroxydammar-(*E*)-20(22)-ene or its isomer	ginsenoside	PN
157	34.30	945.5442	$C_{48}H_{82}O_{18}$	1.4	214.52	–H	945.5424, 783.4902, 621.4373, 459.3838, 179.0547	notoginsenoside K or its isomer	ginsenoside	PN
158[a]	34.32	1107.5957	$C_{54}H_{92}O_{23}$	0.0	268.73	–H	1107.5955, 945.5424, 783.4902, 621.4373, 459.3838, 179.0547	ginsenoside Rb1	ginsenoside	PN
159	36.17	1087.5337	$C_{53}H_{84}O_{23}$	0.6	354.15	–H	1087.5336, 731.4374	lancemasides D or its isomer	ginsenoside	PN

160	36.20	1193.5973	$C_{57}H_{94}O_{26}$	1.0	345.26	–H	1149.6064, 1107.5971, 1089.5821, 945.5417, 783.4882	malonylfloralginsenoside Rb2 or its isomer	ginsenoside	PN
161	36.21	1149.6087	$C_{56}H_{94}O_{24}$	2.2	222.13	–H	1149.6064, 1107.5971, 1089.5821, 945.5417, 783.4882	6''-*O*-acetylginsenoside Rb1 or its isomer	ginsenoside	PN
162	36.36	885.4846	$C_{45}H_{72}O_{17}$	0.4	321.03	+H	885.4815, 723.4322, 577.3721, 415.3174	gracillin or its isomer	steroidal saponin	PS
164	36.42	577.3737	$C_{33}H_{52}O_{8}$	0.3	249.00	+H	577.3739, 415.3202, 271.2051, 253.1941	trillin or its isomer	steroidal saponin	PS
163	36.42	739.4278	$C_{39}H_{62}O_{13}$	2.1	300.25	+H	739.4286, 723.4330, 577.3739, 415.3202, 253.1941	funkioside C or its isomer	steroidal saponin	PS
165	36.44	739.4660	$C_{40}H_{68}O_{12}$	2.9	286.50	–H	739.4665, 475.3808	(20S)-6-*O*-[*β*-D-xylopyranosyl-(1→2)-*β*-D-xylopyranosyl]-dammar-24-ene-3*β*,6*α*,12*β*,20-tetrol or its isomer	ginsenoside	PN
166	36.45	883.4685	$C_{45}H_{72}O_{17}$	–1.3	309.30	–H	883.4704, 737.4079	3-*O*-*β*-D-glucopyranosyl-(1→4)-[*α*-*L*-rhamnopyranosyl(1→2)]-*β*-D-glucopyranosyl-diosgen (PO-3) or its isomer	steroidal saponin	PS
167	36.65	1087.5361	$C_{53}H_{84}O_{23}$	2.8	362.21	–H	1087.5330, 925.4812	lancemasides D or its isomer	ginsenoside	PN
168[a]	37.09	1209.6277	$C_{58}H_{98}O_{26}$	0.2	350.67	–H	1209.6271, 1077.5847, 945.5426, 783.4900, 621.4385	ginsenoside Ra1	ginsenoside	PN
169	37.15	1149.6094	$C_{56}H_{94}O_{24}$	2.7	221.04	–H	1149.6083, 1107.5968, 945.5426, 783.4900	6''-*O*-acetylginsenoside Rb1 or its isomer	ginsenoside	PN
170	37.17	1193.5962	$C_{57}H_{94}O_{26}$	0.1	337.89	–H	1149.6071, 1107.5919, 1089.5854	malonylfloralginsenoside Rb2 or its isomer	ginsenoside	PN
171[a]	37.91	829.4591	$C_{41}H_{68}O_{14}$	0.0	299.65	+HCOO	829.4591	Astragaloside A	ginsenoside	PN

172[a]	37.96	683.4373	$C_{36}H_{62}O_9$	–0.4	269.70	+HCOO	683.4388, 637.4341, 475.3789, 391.2829, 161.0438	20(*S*)-ginsenoside F1	ginsenoside	PN
173[a]	38.37	1077.5853	$C_{53}H_{90}O_{22}$	0.2	352.56	–H	1077.5854, 945.5430, 783.4906, 621.4370	ginsenoside Rb2	ginsenoside	PN
174[a]	38.79	1123.5906	$C_{53}H_{90}O_{22}$	0.0	352.54	+HCOO	1077.5854, 945.5421, 783.4920	ginsenoside Rb3	ginsenoside	PN
175[a]	38.89	925.4792	$C_{47}H_{74}O_{18}$	–1.2	320.36	–H	925.4787, 775.4212, 613.3723, 569.3809	araloside A	ginsenoside	PN
176	39.26	1077.5854	$C_{53}H_{90}O_{22}$	0.3	352.87	–H	1077.5848, 945.5407, 783.4904, 621.4380	notoginsenoside L or its isomer	ginsenoside	PN
177[a]	39.32	943.5270	$C_{48}H_{80}O_{18}$	–0.2	322.72	–H	943.5258, 781.4743	5,6-didehydroginsenoside Rd	ginsenoside	PN
178	39.85	1149.6068	$C_{56}H_{94}O_{24}$	0.5	356.13	–H	1149.6059, 1107.5959, 945.5461, 783.4882	6''-*O*-acetylginsenoside Rb1 or its isomer	ginsenoside	PN
179	40.13	929.5502	$C_{48}H_{82}O_{17}$	2.5	325.50	–H	929.5482, 783.4912, 767.4894, 605.4427	vinaginsenoside R3 or its isomer	ginsenoside	PN
180[a]	40.52	945.5438	$C_{48}H_{82}O_{18}$	1.0	320.00	–H	945.5432, 783.4907, 621.4377, 459.3842, 375.2896, 161.0441	ginsenoside Rd	ginsenoside	PN
181	41.23	1031.5426	$C_{51}H_{84}O_{21}$	–0.6	317.57	–H	987.5538, 945.5430, 783.4910	malonyl-ginsenoside Re or its isomer	ginsenoside	PN
182	41.76	913.4444	$C_{45}H_{70}O_{19}$	0.6	300.59	–H	913.4413, 751.3902	notoginsenoside LX or its isomer	ginsenoside	PN
183	41.87	1175.6267	$C_{58}H_{96}O_{24}$	4.1	360.35	–H	1175.6253, 1107.5982, 945.5420, 783.4918, 621.4366, 459.3834	ginsenoside Ra6 or its isomer	ginsenoside	PN
184	41.89	945.5432	$C_{48}H_{82}O_{18}$	0.4	315.00	–H	945.5420, 783.4918, 621.4366, 459.3834	gypenoside XVII or its isomer	ginsenoside	PN
185	42.05	637.4317	$C_{36}H_{62}O_9$	–0.6	278.11	–H	637.4322, 475.3789	3-*O*-*β*-D-glucopyranosyl-20(*S*)-protopanaxatriol or its isomer	ginsenoside	PN

186	42.05	679.4424	$C_{38}H_{64}O_{10}$	–0.4	279.34	–H	637.4322, 475.3789	6'-acetyl ginsenoside-F1 or its isomer	ginsenoside	PN
187	42.30	915.5305	$C_{47}H_{80}O_{17}$	–1.9	319.83	–H	915.5323, 753.4781, 621.4446	3-*O*-*β*-D-glucopyranosyl-20-*O*-[*α*-*L*-arabinopyranosyl(1→2)-*β*-D-glucopyranosyl]-3β,12β,20(*S*)-trihydroxydammar-24-ene or its isomer	ginsenoside	PN
188	42.81	915.5318	$C_{47}H_{80}O_{17}$	–0.5	320.07	–H	915.5313, 783.4900, 621.4365, 459.3837	3-*O*-*β*-D-glucopyranosyl-20-*O*-[*α*-L-arabinopyranosyl(1→2)-*β*-D-glucopyranosyl]-3*β*,12*β*,20(*S*)-trihydroxydammar-24-ene or its isomer	ginsenoside	PN
189	42.90	797.4696	$C_{41}H_{68}O_{12}$	0.4	294.31	+HCOO	751.6460, 619.4212	notoginsenoside T5 or its isomer	ginsenoside	PN
190	43.37	751.4636	$C_{41}H_{68}O_{12}$	–0.2	299.26	–H	751.4632, 619.4212, 116.9273	notoginsenoside T5 or its isomer	ginsenoside	PN
191[a]	43.78	811.4857	$C_{42}H_{70}O_{12}$	1.0	303.12	+HCOO	765.4792, 619.4202	ginsenoside F4	ginsenoside	PN
192[a]	44.02	665.4267	$C_{36}H_{60}O_{8}$	–0.4	273.40	+HCOO	665.4284, 619.4213, 161.0442	ginsenoside Rk3	ginsenoside	PN
193[a]	44.57	665.4272	$C_{36}H_{60}O_{8}$	0.2	276.11	+HCOO	665.4274, 619.4211, 161.0453	ginsenoside Rh4	ginsenoside	PN
194	44.76	925.4805	$C_{47}H_{74}O_{18}$	0.3	336.15	–H	925.4793, 551.3767, 455.3543	3-*O*-*β*-D-xylopyranosyl-(1→2)-*β*-D-glucopyranosyl-28-*O*-*β*-D-glucopyranosyl oleanolic acid or its isomer	ginsenoside	PN
195	44.96	915.5334	$C_{47}H_{80}O_{17}$	1.2	330.18	–H	915.5328, 783.4905, 621.4370	3-*O*-*β*-D-glucopyranosyl-20-*O*-[α-L-arabinopyranosyl(1→2)-*β*-D-glucopyranosyl]-3*β*,12*β*,20(*S*)-trihydroxydammar-24-ene or its isomer	ginsenoside	PN

196[a]	44.97	829.4954	$C_{42}H_{72}O_{13}$	–0.1	303.83	+HCOO	829.4948, 783.4905, 621.4370	ginsenoside F2	ginsenoside	PN
197	45.20	925.4806	$C_{47}H_{74}O_{18}$	0.4	338.47	–H	925.4806, 731.4372, 569.3852, 455.3515	3-*O*-*β*-D-xylopyranosyl-(1→2)-*β*-D-glucopyranosyl-28-*O*-*β*-D-glucopyranosyl oleanolic acid or its isomer	ginsenoside	PN
198	45.69	313.2373	$C_{18}H_{34}O_4$	–3.5	181.92	–H	313.2387, 295.2257, 183.1382	9,10-dyhydroxy-12-octadecenoic acid or its isomer	organic acid	CP
199	46.13	425.3771	$C_{30}H_{48}O$	–1.7	213.74	+H	425.3771, 257.2268, 189.1633	taraxerone or its isomer	terpenoid	CP
200[a]	46.14	783.4903	$C_{42}H_{72}O_{13}$	0.3	301.53	–H	783.4897, 621.4373, 459.3840, 375.2894, 116.9273	ginsenoside Rg3	ginsenoside	PN
201	46.47	783.4909	$C_{42}H_{72}O_{13}$	1.1	299.10	–H	783.4901, 621.4380, 459.3841, 375.2900, 116.9275	3-*O*-*β*-D-glucopyranosyl-(1→2)-*β*-D-glucopyranoside-12*β*,25-dihydroxydammar-(*E*)-20(22)-ene or its isomer	ginsenoside	PN
202[a]	49.80	765.4800	$C_{42}H_{70}O_{12}$	0.7	307.95	–H	765.4799, 603.4263	ginsenoside Rk1	ginsenoside	PN
203	49.99	235.1682	$C_{15}H_{22}O_2$	–4.6	160.05	+H	235.1687, 179.1048	kanshone I or its isomer	other	NJ
204[a]	50.22	765.4800	$C_{42}H_{70}O_{12}$	0.7	315.04	–H	765.4796, 603.4281, 116.9274	ginsenoside Rg5	ginsenoside	PN
205	50.51	295.2269	$C_{18}H_{32}O_3$	–3.4	179.57	–H	295.2253, 279.2321, 277.2178	9-hydroxy-10,12-octadecadienoic acid or its isomer	organic acid	CP

[a] Components identified with the aid of reference compounds comparison.

Source: CP: Codonopsis Radix; PS: Polygonati Rhizoma; NJ: Nardostachyos Radix et Rhizoma

Table S5. Calibration curves, linearity, LOQ, LOQ, intra-day/inter-day precision, stability, repeatability, recovery, and matrix effect for the UHPLC-sMRM approach targeting to quantify 24 analytes of WXG.

Analytes	Calibration curves	r	Linear range (ng/mL)	LOQ (pg)	LOD (pg)	Concentration	Recovery (n=3, %)	RSD (%)				Matrix effects (%)
								Inter-day	Intra-day	Stability	Repeatability	
syringin	y = 884.2x + 730.6	0.9993	2.5–500	0.63	0.00031	Low	86.6 ± 7.0	2.0	2.8	4.2	9.6	108.1
						Medium	85.7 ± 1.7	3.4	1.5			
						High	88.1 ± 1.8	3.2	1.9			
notoginsenoside R1	y = 5291.9x + 175140.0	0.9968	100–20000	0.098	0.049	Low	85.9 ± 8.0	4.6	4.1	5.7	8.5	92.0
						Medium	92.1 ± 4.3	3.3	2.0			
						High	94.0 ± 4.5	6.5	2.6			
lobetyolin	y = 7924.9x + 13885.9	0.9990	5–1000	0.078	0.039	Low	87.1 ± 10.3	1.2	2.7	3.8	9.3	109.0
						Medium	85.6 ± 2.4	3.6	2.5			
						High	86.0 ± 2.1	3.4	1.5			
ginsenoside Re	y = 3274.1x + 48734.1	0.9978	50–10000	0.049	0.024	Low	90.6 ± 9.8	9.9	7.1	5.3	7.4	80.1
						Medium	92.6 ± 8.1	8.9	3.2			
						High	96.3 ± 5.5	6.0	2.0			
ginsenoside Rg1	y = 1813.9x + 55841.2	0.9957	125–25000	0.49	0.12	Low	91.0 ± 14.6	4.0	4.3	3.6	6.5	83.5
						Medium	86.0 ± 5.3	3.9	1.7			
						High	103.8 ± 13.1	4.6	1.7			
ginsenoside Rf	y = 16558.0x + 6346.3	0.9989	1–200	0.063	0.031	Low	92.1 ± 5.6	2.5	2.6	4.4	9.8	105.9
						Medium	93.6 ± 3.4	2.1	1.4			
						High	85.8 ± 2.4	4.2	1.8			

20(*S*)-notoginsenoside R2	y = 13080.6x + 156512.0	0.9956	25–2500	0.012	0.0061	Low	85.9 ± 6.6	2.9	2.3	3.6	8.2	93.6
						Medium	91.8 ± 3.5	2.6	1.8			
						High	98.6 ± 1.5	4.2	1.5			
notoginsenoside Fa	y = 232.4x + 276.6	0.9985	5–1000	0.63	0.078	Low	99.6 ± 14.6	12.2	5.1	6.0	8.3	85.2
						Medium	97.9 ± 6.3	12.6	2.0			
						High	88.3 ± 11.5	5.4	3.6			
20(*S*)-ginsenoside Rg2	y = 14067.9x + 174596.0	0.9958	25–5000	0.098	0.049	Low	98.3 ± 6.8	2.6	2.7	3.4	8.8	91.8
						Medium	102.6 ± 2.5	2.8	2.1			
						High	106.3 ± 0.9	4.2	1.5			
20(*S*)-ginsenoside Rh1	y = 1424.2x + 13890.2	0.9983	25–5000	0.20	0.0015	Low	99.4 ± 8.9	3.1	2.7	3.3	9.4	102.6
						Medium	100.2 ± 2.1	2.2	2.0			
						High	99.8 ± 1.6	4.5	1.9			
ginsenoside Rb1	y = 856.1x + 11571.9	0.9995	62.5–25000	0.12	0.031	Low	107.8 ± 8.8	11.0	2.4	5.8	8.8	98.2
						Medium	106.3 ± 10.1	8.9	1.0			
						High	109.6 ± 5.5	4.4	2.3			
ginsenoside Ra1	y = 886.5x + 1153.3	0.9994	5–1000	0.31	0.16	Low	85.7 ± 6.8	13.0	4.0	5.8	8.8	85.1
						Medium	85.6 ± 6.6	11.7	3.1			
						High	86.6 ± 7.7	6.0	3.0			
ginsenoside Rb2	y = 1433.9x + 3001.1	0.9992	10–2000	5.00	2.50	Low	94.0 ± 7.9	14.0	3.8	7.7	8.4	85.6
						Medium	89.2 ± 5.5	13.1	2.5			
						High	89.2 ± 4.5	5.1	2.6			
20(*S*)-ginsenoside F1	y = 6977.8x + 25180.3	0.9980	10–2000	0.16	0.020	Low	95.2 ± 9.1	2.4	2.6	3.5	8.1	100.3
						Medium	96.2 ± 1.5	6.3	1.0			
						High	96.8 ± 1.8	3.4	1.7			
araloside A	y = 1949.9x + 1108.4	0.9995	2–400	0.0039	0.0020	Low	93.1 ± 6.4	4.7	3.7	3.2	8.3	104.2

						Medium	95.6 ± 4.0	3.4	2.0			
						High	92.6 ± 8.9	2.9	0.9			
ginsenoside Rd	y = 5681.0x + 206959.0	0.9980	100–10000	0.39	0.20	Low	87.5 ± 3.4	7.4	4.3	3.8	9.0	89.0
						Medium	85.3 ± 4.9	5.8	1.8			
						High	104.3 ± 3.6	4.5	1.5			
ginsenoside F4	y = 18973.0x + 18468.4	0.9985	2.5–500	0.078	0.039	Low	97.8 ± 6.4	1.6	2.7	4.1	8.6	107.1
						Medium	100.9 ± 4.1	4.6	1.7			
						High	98.2 ± 0.5	2.6	1.7			
atractylenolide III	y = 18421.2x + 4850.0	0.9973	1–200	0.13	0.063	Low	112.0 ± 4.9	7.0	3.7	5.4	9.3	108.2
						Medium	100.1 ± 12.1	4.7	5.0			
						High	108.3 ± 7.7	13.1	3.5			
ginsenoside Rk3	y = 1749.9x + 3098.0	0.9983	5–1000	1.25	0.63	Low	100.7 ± 6.0	2.0	2.9	3.9	8.1	103.0
						Medium	100.9 ± 3.3	4.2	1.8			
						High	100.7 ± 1.0	3.9	1.7			
ginsenoside F2	y = 2420.9x + 8505.8	0.9985	10–2000	0.16	0.020	Low	95.3 ± 5.9	11.4	4.7	7.3	7.3	93.59
						Medium	98.0 ± 3.6	10.6	2.3			
						High	98.2 ± 3.7	4.8	1.0			
ginsenoside Rh4	y = 119.2x + 4308.0	0.9985	100–20000	25.00	6.25	Low	98.8 ± 8.3	2.2	3.6	4.0	7.2	101.3
						Medium	95.4 ± 2.2	5.4	1.9			
						High	96.5 ± 2.5	3.4	1.6			
ginsenoside Rg3	y = 15271.2x + 3.8	0.9969	5–1000	0.0049	0.0012	Low	86.5 ± 8.5	2.7	3.7	4.7	8.3	94.9
						Medium	90.7 ± 5.6	1.4	2.0			
						High	92.6 ± 3.4	5.3	2.0			
ginsenoside Rk1	y = 6515.8x + 13914.6	0.9981	5–1000	0.0098	0.0049	Low	95.6 ± 7.2	3.8	4.7	8.1	9.9	85.3
						Medium	100.0 ± 2.6	3.2	1.2			
						High	98.8 ± 3.2	7.5	3.7			

ginsenoside Rg5	y = 14511.8x + 3.7	0.9958	5–1000	0.0049	0.0012	Low	88.2 ± 5.6	3.8	3.5	7.2	8.9	86.2
						Medium	92.6 ± 2.4	2.9	1.0			
						High	93.0 ± 1.3	8.8	2.7			

Table S6. The contents of 24 analytes in 27 batches of WXG samples (mg/bag).

Analytes	Batch No.																										
	**1**	**2**	**3**	**4**	**5**	**6**	**7**	**8**	**9**	**10**	**11**	**12**	**13**	**14**	**15**	**16**	**17**	**18**	**19**	**20**	**21**	**22**	**23**	**24**	**25**	**26**	**27**
syringin	0.08	0.15	0.12	0.06	0.12	0.10	0.11	0.11	0.09	0.13	0.08	0.06	0.08	0.15	0.08	0.07	0.06	0.06	0.05	0.05	0.06	0.05	0.05	0.06	0.07	0.08	0.06
noto-R1*	2.08	2.28	2.59	3.30	2.46	2.57	2.87	2.88	2.51	3.45	2.90	3.02	3.24	3.87	2.60	2.27	3.12	3.01	3.14	3.00	2.38	3.37	3.23	3.30	3.29	3.59	3.63
lobetyolin	0.14	0.20	0.20	0.12	0.23	0.12	0.14	0.14	0.13	0.17	0.15	0.11	0.17	0.19	0.13	0.08	0.10	0.10	0.09	0.08	0.09	0.07	0.10	0.10	0.11	0.12	0.13
Re	0.91	0.89	1.01	1.23	0.99	0.98	1.10	1.05	1.00	1.32	1.07	1.15	1.23	1.52	1.01	0.93	1.19	1.11	1.19	1.13	0.97	1.30	1.23	1.26	1.24	1.37	1.42
Rg1*	12.82	12.86	14.81	16.56	14.02	14.40	15.13	15.03	13.83	17.30	14.66	15.36	16.08	18.83	12.74	11.61	13.71	13.34	13.48	13.40	11.83	14.39	13.88	14.24	14.18	15.38	15.59
Rf	0.02	0.02	0.02	0.02	0.02	0.02	0.02	0.02	0.02	0.02	0.02	0.02	0.02	0.03	0.02	0.02	0.02	0.02	0.02	0.02	0.02	0.02	0.02	0.02	0.02	0.02	0.03
20(*S*)-noto-R2	0.36	0.40	0.45	0.51	0.38	0.43	0.46	0.47	0.44	0.54	0.46	0.42	0.47	0.58	0.46	0.40	0.51	0.48	0.46	0.45	0.38	0.53	0.52	0.49	0.53	0.56	0.59
noto-Fa	0.26	0.22	0.27	0.28	0.24	0.26	0.26	0.26	0.25	0.31	0.24	0.26	0.26	0.33	0.26	0.23	0.30	0.24	0.26	0.27	0.24	0.32	0.29	0.31	0.32	0.36	0.38
20(*S*)- Rg2	0.31	0.32	0.36	0.38	0.31	0.35	0.36	0.37	0.34	0.42	0.34	0.33	0.36	0.45	0.36	0.33	0.39	0.35	0.34	0.35	0.32	0.39	0.38	0.38	0.39	0.42	0.43
20(*S*)- Rh1	0.52	0.62	0.61	0.62	0.47	0.59	0.63	0.68	0.66	0.68	0.58	0.49	0.58	0.70	0.65	0.61	0.66	0.58	0.53	0.55	0.51	0.60	0.69	0.54	0.72	0.65	0.78
Rb1*	8.98	8.73	10.57	11.89	9.45	10.48	10.31	10.57	9.90	12.28	10.53	10.45	10.90	13.77	10.43	8.92	11.89	9.86	10.12	10.69	9.38	11.95	11.81	11.97	12.43	13.49	13.63
Ra1	0.03	0.03	0.04	0.05	0.03	0.03	0.03	0.03	0.04	0.05	0.04	0.05	0.05	0.06	0.04	0.03	0.05	0.04	0.04	0.04	0.03	0.05	0.05	0.05	0.06	0.06	0.06
Rb2	0.07	0.08	0.12	0.14	0.09	0.09	0.09	0.09	0.11	0.14	0.13	0.13	0.14	0.16	0.12	0.10	0.16	0.15	0.14	0.14	0.10	0.16	0.15	0.16	0.16	0.17	0.18
20(*S*)-F1	0.07	0.05	0.07	0.08	0.05	0.06	0.06	0.06	0.07	0.07	0.06	0.06	0.07	0.08	0.07	0.06	0.08	0.07	0.07	0.07	0.06	0.08	0.08	0.08	0.09	0.09	0.10
chikusetsusaponin IV	0.01	0.01	0.01	0.02	0.01	0.02	0.02	0.02	0.02	0.02	0.02	0.02	0.02	0.02	0.02	0.01	0.02	0.02	0.02	0.02	0.01	0.02	0.02	0.02	0.02	0.02	0.02
Rd	1.61	1.56	1.76	2.28	1.70	1.93	1.92	2.00	1.97	2.42	2.06	2.15	2.33	2.70	2.16	1.85	2.45	2.27	2.24	2.31	1.86	2.49	2.43	2.51	2.59	2.76	2.82
F4	0.02	0.03	0.03	0.02	0.01	0.03	0.03	0.03	0.03	0.03	0.03	0.02	0.02	0.03	0.04	0.04	0.04	0.03	0.03	0.03	0.02	0.03	0.04	0.02	0.04	0.03	0.04
atractylenolide III	0.01	0.01	0.01	0.01	0.01	0.01	0.01	0.01	0.01	0.01	0.01	0.01	0.01	0.01	0.01	0.01	0.01	0.01	0.01	0.01	0.01	0.01	0.01	0.01	0.01	0.01	0.01
Rk3	0.07	0.11	0.09	0.08	0.05	0.09	0.09	0.11	0.12	0.11	0.09	0.06	0.08	0.10	0.14	0.13	0.15	0.12	0.10	0.10	0.07	0.12	0.15	0.09	0.16	0.11	0.16
F2	0.04	0.03	0.03	0.04	0.03	0.04	0.03	0.03	0.03	0.03	0.03	0.03	0.03	0.03	0.04	0.04	0.03	0.03	0.03	0.04	0.04	0.04	0.05	0.04	0.05	0.05	0.05
Rh4	1.71	3.00	2.35	2.14	1.25	2.31	2.36	2.83	3.01	2.69	2.31	1.44	2.07	2.63	3.17	3.13	3.27	2.64	2.13	2.23	1.74	2.62	3.46	1.99	3.54	2.59	3.69

Rg3	0.12	0.20	0.16	0.17	0.10	0.14	0.17	0.19	0.21	0.19	0.21	0.14	0.19	0.18	0.19	0.18	0.17	0.14	0.13	0.13	0.11	0.15	0.19	0.12	0.19	0.15	0.19
Rk1	0.06	0.11	0.08	0.08	0.04	0.08	0.09	0.10	0.11	0.10	0.10	0.06	0.09	0.09	0.13	0.14	0.13	0.10	0.10	0.10	0.06	0.12	0.14	0.09	0.14	0.12	0.15
Rg5	0.06	0.11	0.09	0.08	0.05	0.08	0.09	0.10	0.11	0.10	0.10	0.07	0.09	0.10	0.11	0.12	0.12	0.09	0.09	0.09	0.06	0.10	0.12	0.08	0.13	0.10	0.12
sum of CP marks	23.87	23.88	27.97	31.75	25.93	27.45	28.30	28.48	26.23	33.03	28.09	28.83	30.22	36.47	25.77	22.80	28.72	26.21	26.74	27.09	23.60	29.71	28.92	29.51	29.90	32.46	32.84

*Three ginsenosides recorded by the Chinese Pharmacopoeia, 27 batches of WXG were qualified.
